# Novel roles of an intragenic G-quadruplex in controlling microRNA expression and cardiac function

**DOI:** 10.1093/nar/gkab055

**Published:** 2021-02-09

**Authors:** Min Zhu, Juan Gao, Xian-Juan Lin, Yun-Yun Gong, Yan-Chao Qi, Yuan-Liang Ma, Yuan-Xiu Song, Wei Tan, Fang-Yuan Li, Min Ye, Jun Gong, Qing-Hua Cui, Zeng-Hui Huang, You-Yi Zhang, Xiu-Jie Wang, Feng Lan, Shi-Qiang Wang, Gu Yuan, Yue Feng, Ming Xu

**Affiliations:** Department of Cardiology, Institute of Vascular Medicine, NHC Key Laboratory of Cardiovascular Molecular Biology and RegulatoryPeptides, Key Laboratory of Molecular Cardiovascular Science, Ministry of Education, Peking University Third Hospital, Beijing 100191, China; Department of Cardiology, Institute of Vascular Medicine, NHC Key Laboratory of Cardiovascular Molecular Biology and RegulatoryPeptides, Key Laboratory of Molecular Cardiovascular Science, Ministry of Education, Peking University Third Hospital, Beijing 100191, China; Department of Cardiology, Institute of Vascular Medicine, NHC Key Laboratory of Cardiovascular Molecular Biology and RegulatoryPeptides, Key Laboratory of Molecular Cardiovascular Science, Ministry of Education, Peking University Third Hospital, Beijing 100191, China; State Key Laboratory of Biomembrane and Membrane Biotechnology, College of Engineering and College of Life Sciences, Peking University, Beijing 100871, China; Department of Cardiology, Institute of Vascular Medicine, NHC Key Laboratory of Cardiovascular Molecular Biology and RegulatoryPeptides, Key Laboratory of Molecular Cardiovascular Science, Ministry of Education, Peking University Third Hospital, Beijing 100191, China; Department of Cardiology, Institute of Vascular Medicine, NHC Key Laboratory of Cardiovascular Molecular Biology and RegulatoryPeptides, Key Laboratory of Molecular Cardiovascular Science, Ministry of Education, Peking University Third Hospital, Beijing 100191, China; Department of Cardiology, Institute of Vascular Medicine, NHC Key Laboratory of Cardiovascular Molecular Biology and RegulatoryPeptides, Key Laboratory of Molecular Cardiovascular Science, Ministry of Education, Peking University Third Hospital, Beijing 100191, China; Department of Chemical Biology, College of Chemistry, Peking University, Beijing 100871, China; Department of Chemical Biology, College of Chemistry, Peking University, Beijing 100871, China; State Key Laboratory of Natural and Biomimetic Drugs, Peking University, Beijing 100191, China; College of Life Sciences, Institute of Model Animal of Wuhan University, Wuhan 430072, China; Department of Biomedical Informatics, School of Basic Medical Sciences, Center for Noncoding RNA Medicine, Peking University, Beijing 100191, China; Key Laboratory of Genetic Network Biology, Institute of Genetics and Developmental Biology, Chinese Academy of Sciences, Beijing 100101, China; Department of Cardiology, Institute of Vascular Medicine, NHC Key Laboratory of Cardiovascular Molecular Biology and RegulatoryPeptides, Key Laboratory of Molecular Cardiovascular Science, Ministry of Education, Peking University Third Hospital, Beijing 100191, China; Key Laboratory of Genetic Network Biology, Institute of Genetics and Developmental Biology, Chinese Academy of Sciences, Beijing 100101, China; Beijing Lab for Cardiovascular Precision Medicine, Anzhen Hospital, Capital Medical University, Beijing 10029, China; State Key Laboratory of Biomembrane and Membrane Biotechnology, College of Engineering and College of Life Sciences, Peking University, Beijing 100871, China; Department of Chemical Biology, College of Chemistry, Peking University, Beijing 100871, China; Department of Pharmacology and Chemical Biology, Emory University School of Medicine, Atlanta, GA 30322, USA; Department of Cardiology, Institute of Vascular Medicine, NHC Key Laboratory of Cardiovascular Molecular Biology and RegulatoryPeptides, Key Laboratory of Molecular Cardiovascular Science, Ministry of Education, Peking University Third Hospital, Beijing 100191, China; State Key Laboratory of Natural and Biomimetic Drugs, Peking University, Beijing 100191, China

## Abstract

Simultaneous dysregulation of multiple microRNAs (miRs) affects various pathological pathways related to cardiac failure. In addition to being potential cardiac disease-specific markers, miR-23b/27b/24-1 were reported to be responsible for conferring cardiac pathophysiological processes. In this study, we identified a conserved guanine-rich RNA motif within the miR-23b/27b/24-1 cluster that can form an RNA G-quadruplex (rG4) *in vitro* and in cells. Disruption of this intragenic rG4 significantly increased the production of all three miRs. Conversely, a G4-binding ligand tetrandrine (TET) stabilized the rG4 and suppressed miRs production in human and rodent cardiomyocytes. Our further study showed that the rG4 prevented Drosha-DGCR8 binding and processing of the pri-miR, suppressing the biogenesis of all three miRs. Moreover, CRISPR/Cas9-mediated G4 deletion in the rat genome aberrantly elevated all three miRs in the heart *in vivo*, leading to cardiac contractile dysfunction. Importantly, loss of the G4 resulted in reduced targets for the aforementioned miRs critical for normal heart function and defects in the L-type Ca^2+^ channel-ryanodine receptor (LCC-RyR) coupling in cardiomyocytes. Our results reveal a novel mechanism for G4-dependent regulation of miR biogenesis, which is essential for maintaining normal heart function.

## INTRODUCTION

Cardiovascular disease (CVD) is a leading cause of mortality worldwide, of which, heart failure (HF) is the main cause of death. Growing evidence supports that numerous microRNAs (miRs) are dysregulated upon insult to cardiomyocytes, which affects the functional network of target genes involved in various pathological steps toward heart failure (1,2). Deciphering the molecular mechanisms underlying the deleterious co-regulation of miRs that contribute to heart impairment is challenging. Interestingly, a number of miRs that play important roles in cardiomyocytes are derived from primary transcripts encoding a few clusters of miRs, such as the miR-221/222 cluster, which balances the antiviral and inflammatory response in viral myocarditis, and the miR-17–92 cluster, which is involved in cardiovascular development and cardiovascular diseases (3,4). Another example is miR-24, which is processed along with miR-23 and miR-27 from pri-miR clusters (5), whose aberrant increase causes cardiac malfunction (6,7). However, the mechanisms that regulate mature miR production from miR-clusters remains poorly understood.

G-quadruplexes (G4s) are non-canonical nucleic acid secondary structures that form within guanine-rich strands widely distributed in gene promoters (8), introns (9) and at the chromosome ends (10,11), which play important roles in regulation, alternative splicing and chromosomal stability. Our previous studies discovered a guanine-rich (G-rich) nucleic acid sequence motif within the miR-23b/27b/24-1 cluster that can form a DNA G4 *in vitro* (5). Although increasing lines of evidence have shown that G4s function in gene regulation and various disease conditions (12,13), whether and how the G4 in the miR-23b/27b/24-1 cluster may govern the biogenesis of neighboring miRs and heart function is an intriguing question that remains to be answered.

In the present study, using a combination of chemical, molecular biology, genome editing and pharmacological approaches, we discovered the essential function of this conserved G4 in suppressing miR biogenesis from the miR-23b/27b/24-1 cluster in human and rat cardiomyocytes. Mechanistically, the G4 acts by preventing the Drosha-DGCR8 microprocessor from binding to the primary transcript of the miR cluster. Moreover, we identified a molecular network and functional target genes downstream of the G4–miR pathway. Finally, we demonstrated that the disruption of G4-dependent miR regulation leads to defective E–C coupling and contractile dysfunction.

## MATERIALS AND METHODS

### Circular dichroism spectrometry (CD)

The CD experiments were performed using a J-815 CD spectrometer (JASCO, Japan). The oligonucleotides (Supplementary Table S1) were diluted to 10 μmol/l in 0–150 mmol/l KCl and 30 mmol/l Tris–HCl buffer (pH7.4). All samples were annealed at 95°C for 10 min and slowly cooled to 4°C during a period of at least 8 h before use. The samples were scanned in a 1.0-cm-path-length cuvette three times to obtain the average spectrum from 200 to 400 nm (14).

### Nuclear magnetic resonance (NMR)

The NMR experiments were carried out with a Bruker AV700 spectrometer (700 MHz) at room temperature. The samples were dissolved and annealed in 25 mmol/l K_2_HPO_4_–KH_2_PO_4_ (pH 7.0) and 100 mmol/l KCl with 10% D_2_O to a final concentration of 1mmol/l. The 1-D^1^H NMR spectrum was collected (15).

### RNase T1 footprinting

RNA G-rich sequence (Supplementary Table S1) was labelled by 6-FAM at the 3′ end. The FAM-labeled samples were diluted with 60 mmol/l Tris–HCl buffer (pH 7.4) to 0.1 μmol/l while with no salts, 150 mmol/l LiCl or 150 mmol/l KCl; afterward, the samples were annealed at 95°C and then slowly cooled to 4°C. 0.1 μmol/l 6-FAM labeled samples were subjected to RNase T1 digestion at 37°C for 15 min and then quenched on ice. The samples were resolved by 20% polyacrylamide gel at 1500 V for 4 h at 4°C before been imaged with a GE Healthcare Typhoon9500 gel scanner (12).

### Visualization of RNA G4 experiment in cells

Visualization of the RNA G4 experiment in cells (Supplementary Figure S1A) was performed as described previously (16,17). ISCH-oa1 (a gift from Professor Jia-Heng Tan from Sun Yat-Sen University) and azido-modified-anti-G4 tail oligonucleotide (a 25 bases oligonucleotide complementary to the oligonucleotide sequence that is adjacent (tail sequence) to the 3′ end of the G-rich sequence) (synthesized by TaKaRa, Dalian, China) (Supplementary Figure S1B and Supplementary Table S1) were mixed in DEPC-treated water (DEPC-H_2_O), containing fresh sodium ascorbate and copper sulphate for 24 h at 37°C. RP-HPLC-UV and mass spectrometry were used to determine the purity of the new synthetic. HEK293A cells were grown in Dulbecco's modified Eagle's medium (DMEM) supplemented with 10% fetal bovine serum (FBS) and 100 U/ml penicillin/streptomycin at 37°C, with 5% CO_2_ atmosphere. The cells were then seeded in a confocal dish (Corning, USA) and grown overnight. RNA oligonucleotides labelled with 6-FAM at the 3′ end (Supplementary Table S1) were transfected into HEK293A cells using Lipofectamine 2000 (Invitrogen, USA) for 6 h. Cells were fixed with 4% paraformaldehyde in DEPC–PBS for 15 min. After washing in DEPC-PBS thrice, cells were permeabilized in 0.5% Triton X-100/DEPC/PBS at room temperature for 15 min. After washing with 2 × SSC, probes-anti-G4 tail sequence or Cy5-anti-G4 tail sequence (Supplementary Figure S1B and Supplementary Table S1) was diluted at 0.3M in hybridization buffer (4 × SSC, 0.5 mM EDTA, 10% dextran sulfate, 30% deionized-formamide in DEPC-H_2_O) and applied to the cells. After hybridization at 37°C overnight, cells were washed twice in 2× SSC for 15 min, followed by DAPI staining for 10 min at 37°C. After washing the cells in DEPC-PBS for 15 min thrice, they were studied using a laser confocal microscope (Carl Zeiss, LSM-780, Germany).

### Electrospray ionization mass spectroscopy (ESI-MS)

Normal ESI mass spectra were performed on a Finnigan LCQ Deca XP Plus ion trap mass spectrometer (San Jose, CA, USA). The direct infusion flow-rate was 2.0 μl/min. The instrument was used in the negative ion mode, with the capillary voltage set to 2.7 kV spray voltage and a 120°C capillary temperature. During all experiments, the scanned mass range was 500–2000. The small molecules (TET) were each directly dissolved in methanol, generating a 1 mmol/l stock solution. The single-stranded RNA G-rich sequence was directly dissolved in a mixture containing 25% methanol, 25% ammonium acetate solution (150 mmol/l in final), and 50% ddH_2_O to a final concentration of the oligonucleotides was 10 μmol/l (18).

### Cell culture and treatments

Rat neonatal cardiomyocytes (NRCMs) and HEK293A cells were grown in DMEM supplemented with 10% FBS and 100 U/ml penicillin/streptomycin. Adenoviruses harboring G-rich sequence (5′- GAGGGTGGGGTTGGGGGTGG -3′), or G-rich sequence deleted (5′-GAGGTGGGGTTG-3′), or with guanine mutated as underlined (5′- GAGTTTGTTGTTGGTTGTTT -3′) were used individually to infect cardiomyocytes at an optimized MOI. All adenoviruses remained in the culture medium for the duration of the experiment. In the tetrandrine (TET) treatments assays, 10^−8^, 10^−7^, 10^−6^ mol/l of TET were added to cell culture medium for 12 h (with adenoviruses) or 24 h (without adenoviruses), respectively. Human embryonic stem cell-derived cardiomyocytes (hESCs-CMs) were cultured in RPMI 1640 medium (Corning) with B-27 Supplement (Gibco, USA), and TET treatment process as above.

### Cardiac differentiation of human embryonic stem cells (hESCs)

HESCs-H9 were purchased from the Cellapy Biological Technology Company (Beijing, China). HESCs-H9 were cultured and differentiation into cardiomyocytes following procedures described previously (19,20). Briefly, hESCs were cultured on six-well plates (Corning) with PSCeasy hESCs culture medium (Cellapy, Beijing, China). Cells were grown to reach 80–90% confluence prior to cardiomyocyte differentiation as described previously (21). The purity of human cardiomyocytes using a purification medium RPMI 1640 (no glucose) (Corning) was monitored by immunofluorescent staining with primary antibodies against TNNT2 (Santa Cruz, USA) and α-actinin (Abcam, USA).

### Detection of mRNA, pri-miR, pre-miR and mature miR

Total RNA was extracted from rat cardiac tissues and cultured cells using Trizol reagent (Invitrogen, USA). Genomic DNA was removed using DNA-free Kit (Invitrogen). First-strand cDNA was synthesized using oligo(dT)15 and SuperScript III Reverse Transcriptase (Invitrogen). One μg cDNA was used for real-time PCR (qPCR) amplification with TransStart Tip Green qPCR SuperMix (Transgen Biotech, China), and GAPDH mRNA was used as the internal control. For miR, pri-miR and pre-miR quantification, total RNA was reverse-transcribed using the miRcute miR First-Strand cDNA Synthesis Kit (Tiangen, China), High Capacity RNA-to-cDNA Kit (Applied Biosystems, USA), the miScript II RT Kit (Qiagen, USA), respectively. Quantitative real-time PCR for miR, pri-miR, or pre-miR was performed using the miRcute Plus miR qPCR Kit (Tiangen), Taqman Fast Universal PCR Master Mix (Applied biosystem, USA), or miScript SYBR Green PCR Kit (Qiagen), respectively. The fluorescent signals were monitored by QuantStudio^®^ 3 Real-Time PCR System (Applied Biosystems). The small nuclear RNA U6 was used as the internal control for miR samples, GAPDH was used as the control for mRNA and pri-miR quantification and pre-U6 was used as a control for pre-miR quantification. All primers used in this study are listed in Supplementary Table S2.

### RNA immunoprecipitation

RNA immunoprecipitation (RIP) experiments were performed using an RIP kit (17-701, Millipore, USA) according to the manufacturer's instructions. Briefly, 1 × 10^7^ HEK293A cells were plated on 10-cm dishes and infected using adenoviruses expressing wild-type (WT) miR cluster, G4 deletion (DEL) or G4 mutation (MUT), followed by TET (10^−6^ mol/l) treatment for 12 h. Cells were lysed with RIP lysis buffer. The lysate was preincubated with magnetic beads at room temperature for 30 min, followed by immunoprecipitation using antibody against Drosha (ab12286, Abcam, UK) or DGCR8 (ab191875, Abcam) overnight at 4°C. IgG was used as the negative control, and anti-SNRNP70 was used as the positive control. After washing, immunoprecipitated proteins were digested with Proteinase K at 55°C for 30 min, and RNA was extracted and purified with phenol: chloroform: isoamyl alcohol. cDNA synthesis and PCR or real-time PCR were performed using the primers listed in Supplementary Table S2.

### Animals

The wild type Sprague Dawley (SD) rats were obtained from K&D Biotechnology Inc (Wuhan, China). Deletion of the core sequence (TGGGGTTGGGG) in the G4 located in the miR-23b/27b/24-1 cluster in SD rats were achieved by CRISPR/cas9 technique in K&D Biotechnology Inc. Whole-genome sequencing was performed at BGI-shenzhen, which confirmed the deletion of the targeted sequence and no additional disruption in the genome. All experimental protocols were approved by the Peking University Institutional Committee for Animal Care and Use (LA 2016-145).

### Whole cell patch clamp combined with laser confocal imaging

The extracellular solution for recording ventricular myocytes contained (in mM): NaCl 135, KCl 4.0, CaCl_2_ 1.0, MgCl_2_ 1.2, NaH_2_PO_4_ 1.2, Glucose 10, HEPES 10, 0.02 tetrodotoxin, pH7.3–7.4. The pipette solution contained (in mM): 110 CsCl, 6 MgCl_2_, 5 Na_2_ATP, 15 TEA-Cl, 10 HEPES and 0.2 fluo-4 pentapotassium, pH7.2. Calcium current was activated via depolarization pulses ranging from –70 to +70 mV in 10-mV increments at 10-s intervals using an EPC7 amplifier (List Medical Electronic, Germany). Intracellular Ca^2+^ dynamic imaging was recorded using an inverted confocal microscope (LSM-510, Carl Zeiss, Germany). Line-scan images were acquired at 3.84 ms/line for whole-cell recording. The Ca^2+^ concentration was reported as the fluorescence ratio according the equation: *R* = (*F* – *F*_0_)/*F*_0_, where *F* is the peak fluorescence and *F*_0_ is the fluorescence at resting state.

### Statistical analysis

Statistical analyses were performed with SPSS 19.0 (IBM Corp, USA) and GraphPad Prism 5.0 (GraphPad Prism Software Inc., USA). All data are presented as mean ± SEM, with the SEM indicated by the error bars. Shapiro-Wilk was used to test the normal distribution in continuous data, and Levene's Test was used to detect the equality of variances. Normal distribution was presented as mean ± the standard error, and the Student's t-test for independent samples was used to detect the differences between groups. Mann–Whitney test was employed for the nonparametric analysis. *P* < 0.05 was considered as statistically significant.

## RESULTS

### G-rich sequence motif in the miR-23b/27b/24-1 cluster forms a G4 that negatively regulates three miRs and pre-miRs biogenesis

**Figure 1.**
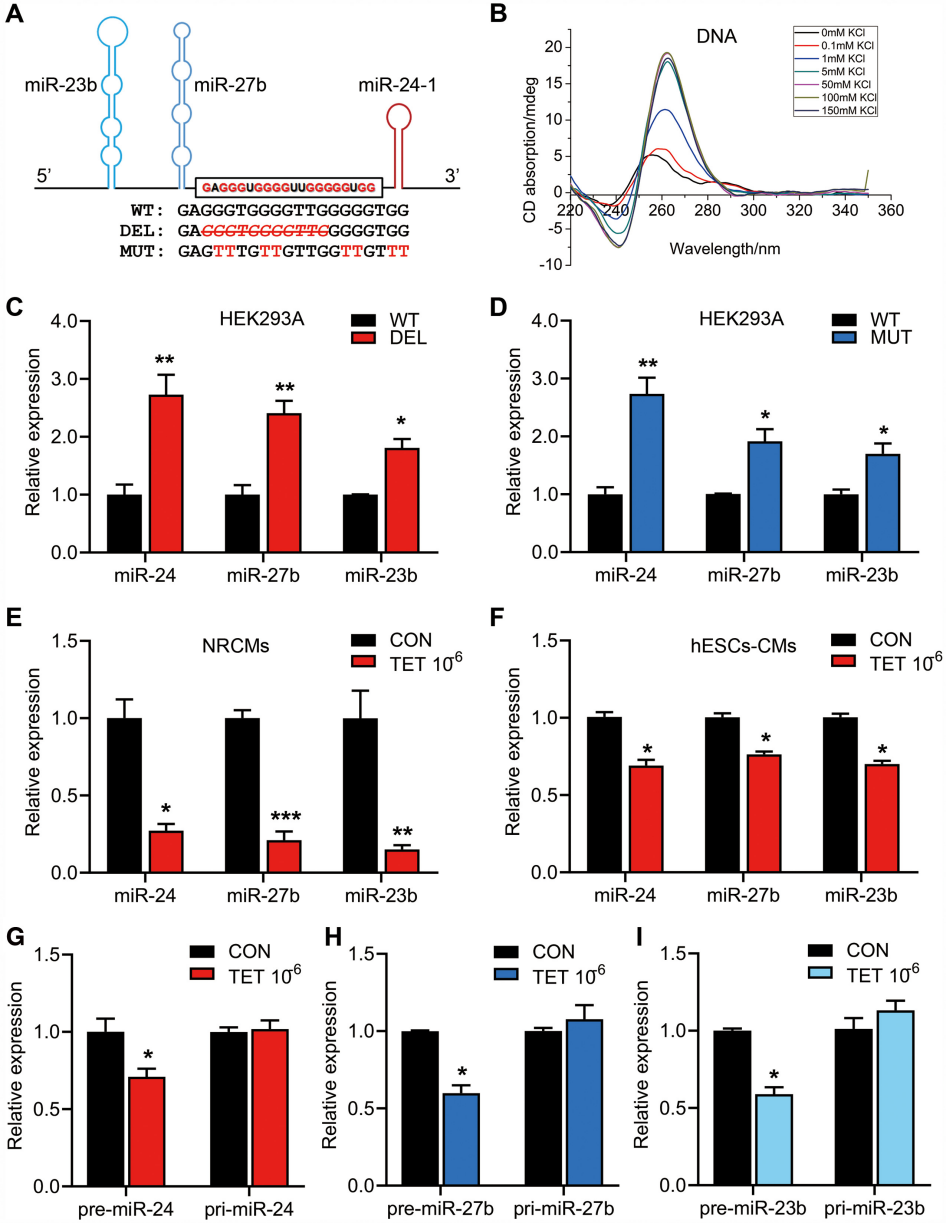
Formation of DNA G4 and increased miR production caused by G4 deletion or mutation, and stabilization of the G4 by TET-mediated down-regulation of miR-24/miR-27b/miR-23b and pri-miR expression. (**A**) Position of the G-rich sequence in the rat miR-23b/27b/24-1 cluster. Wild-type adenovirus (WT-ADV), deleted-type adenovirus (DEL-ADV) and mutated-type adenovirus (MUT-ADV) construction. The crossed italic letter with the red color represents the deleted bases, and mutated bases are marked in red. (**B**) CD absorptivity shows characteristic K^+^-dependent spectra of DNA G4. (**C**) RT-qPCR for quantifying miR-24, miR-27b and miR-23b in HEK293A cells transduced with WT-ADV and DEL-ADV after 24 h. (**D**) RT-qPCR for quantifying miR-24, miR-27b and miR-23b in HEK293A cells transduced with WT-ADV and MUT-ADV after 24 h. (E and F) RT-qPCR for detecting miR-24, miR-27b and miR-23b expression in the NRCMs (**E**) or hESCs-CMs (**F**) treated with TET (10^−6^ mol/l) for 24 h. (**G**) RT-qPCR for detecting pre-miR-24-1 and pri-miR-24-1 expression of NRCMs treated with TET (10^−6^ mol/l) for 24 h. (**H**) RT-qPCR for detecting pre-miR-27b and pri-miR-27b expression in the NRCMs treated with TET (10^−6^ mol/l) for 24 h. (**I**) RT-qPCR for detecting pre-miR-23b and pri-miR-23b expression in the NRCMs treated with TET (10^−6^ mol/l) for 24 h. Graphs show mean ± SEM of three independent experiments (*P < 0.05; **P < 0.01; ***P < 0.001).

Our previous study indicated that a G-rich sequence motif (5′-GAGGGTGGGGGGTTGGGGGTGG-3′) located 133 bp upstream of rat miR-24-1 in the miR-23b/27b/24-1 cluster could form a DNA G4 *in vitro* (Figure 1A) (5). The circular dichroism (CD) spectroscopy revealed that the G-rich DNA sequence showed K^+^-dependent formation of G4, displaying a positive peak at 260 nm and a negative peak at 240 nm (Figure 1B), which indicated a characteristic spectrum for parallel G4 formation (22). Moreover, CD spectrometry verified that the G-rich sequence deletion or mutation abolished the characteristic absorption peak for the G4 (Supplementary Figure S2A and B), reflecting the ability of this G-rich sequence to form the G4 structure. Furthermore, ^1^H nuclear magnetic resonance (NMR) spectrum of the DNA G-rich sequences in 100 mmol/l KCl solution displayed peaks of partially resolved imino protons at δ10.8–12.0 ppm, suggesting the formation of a three-layer G4 (Supplementary Figure S2C). In addition, dimethyl sulfate (DMS) footprinting assay showed that G1, G3–5, G7–9, G15–17 and G19–20 participated in the DNA G4 formation, as indicated by protection of these guanosines against DMS methylation and piperidine cleavage in K^+^ solution (Supplementary Figure S2D). Notably, the aforementioned G-rich sequence motif in the rat genome is conserved in humans (Supplementary Figure S3A). The CD spectra showed that the human G-rich sequence also formed a G4 structure at the DNA level (Supplementary Figure S3B).

To elucidate the biological effects of this G4 within the miR-23b/27b/24-1 cluster on miR expression, we established adenoviral constructs (ADV) that express the wild-type (WT) miR cluster containing the intact G4, and the mutant miR cluster with partial deletion of the G4 (DEL) (Figure 1A). When expressed in HEK293A cells, loss of the G4 in the DEL construct caused a significant increase in all miRs derived from the cluster as compared to that caused by the loss of G4 in the WT construct (Figure 1C). To further verify the function of G4 in the biogenesis of the aforementioned miRs, we mutated guanosines that are critical for G4 formation in the miR-cluster ADV construct (MUT, Figure 1A). Consistent with the finding of DEL-ADV, the abundance of miR-24, miR-27b and miR-23b produced from the MUT-ADV construct was significantly increased compared to that in WT-ADV in HEK293A cells (Figure 1D). These results demonstrated that the G4 negatively regulates the production of miRs encoded by the miR-23b/27b/24-1 cluster.

To further verify the down-regulation effect of G4 on miRs expression, we next used tetrandrine (TET), which has a selective binding affinity to the G4 and stabilized it significantly (5), to test whether pharmacological stabilization of the G4 may further reduce the expression of three miRs. Intriguingly, exposing NRCMs to TET (10^−6^ mol/l) for 24 h resulted in a significant reduction of all three miRs (Figure 1E). Furthermore, we used human embryonic stem cells derived cardiomyocytes (hESCs-CMs) (Supplementary Figure S4) to evaluate the effect of TET on regulating the expression of the aforementioned miRs (Figure 1F). TET (10^−8^, 10^−7^, 10^−6^ mol/l) elicited a dose-dependent reduction of miR-24, miR-27b, and miR-23b in hESCs-CMs (Supplementary Figure S5A), consistent with the idea that the conserved G4 mediates the pharmacological effects of TET in controlling miR expression.

To further investigate whether TET down-regulates the aforementioned miRs through stabilizing the intragenic G4 located in the pri-miR, HEK293A cells were transduced with WT-ADV, DEL-ADV and MUT-ADV for 24 h, followed by TET treatment (10^−8^, 10^−7^, 10^−6^ mol/l) for 12 h. A dose-dependent reduction of miR-24 was observed from the WT-ADV construct. In contrast, miR-24 expression from the DEL-ADV or MUT-ADV was not affected by TET (Supplementary Figure S5B). Interestingly, miR expression from another miR-cluster, rat and human pri-miR-23a/27a/24-2 (without intragenic RNA G-rich sequences) (Supplementary Figure S6), was not affected in the NRCMs and hESCs-CMs cells treated with TET (10^−6^ mol/l) (Supplementary Figure S6). These data suggested that TET down-regulates miR-24, miR-27b and miR-23b specifically from the miR-23b/27b/24-1 cluster by stabilizing the intragenic G4.

Simultaneously, we tested the effect of TET on the biogenesis of pri-miRs and pre-miRs derived from the miR-23b/27b/24-1 cluster. Interestingly, upon TET-treatment (10^−6^ mol/l), expression levels of pre-miR-24, pre-miR-27b and pre-miR-23b were markedly reduced (Figure 1G-H) without affecting the corresponding pri-miRs. These results suggested that the effect of TET on down-regulation of miR-23b, miR-27b, and miR-24 expression was elicited during the generation of pre-miR from pri-miR, prompting further discussion.

### The G-rich sequence within miR-23b/27b/24-1 forms an intragenic RNA G4 stabilized by TET

**Figure 2.**
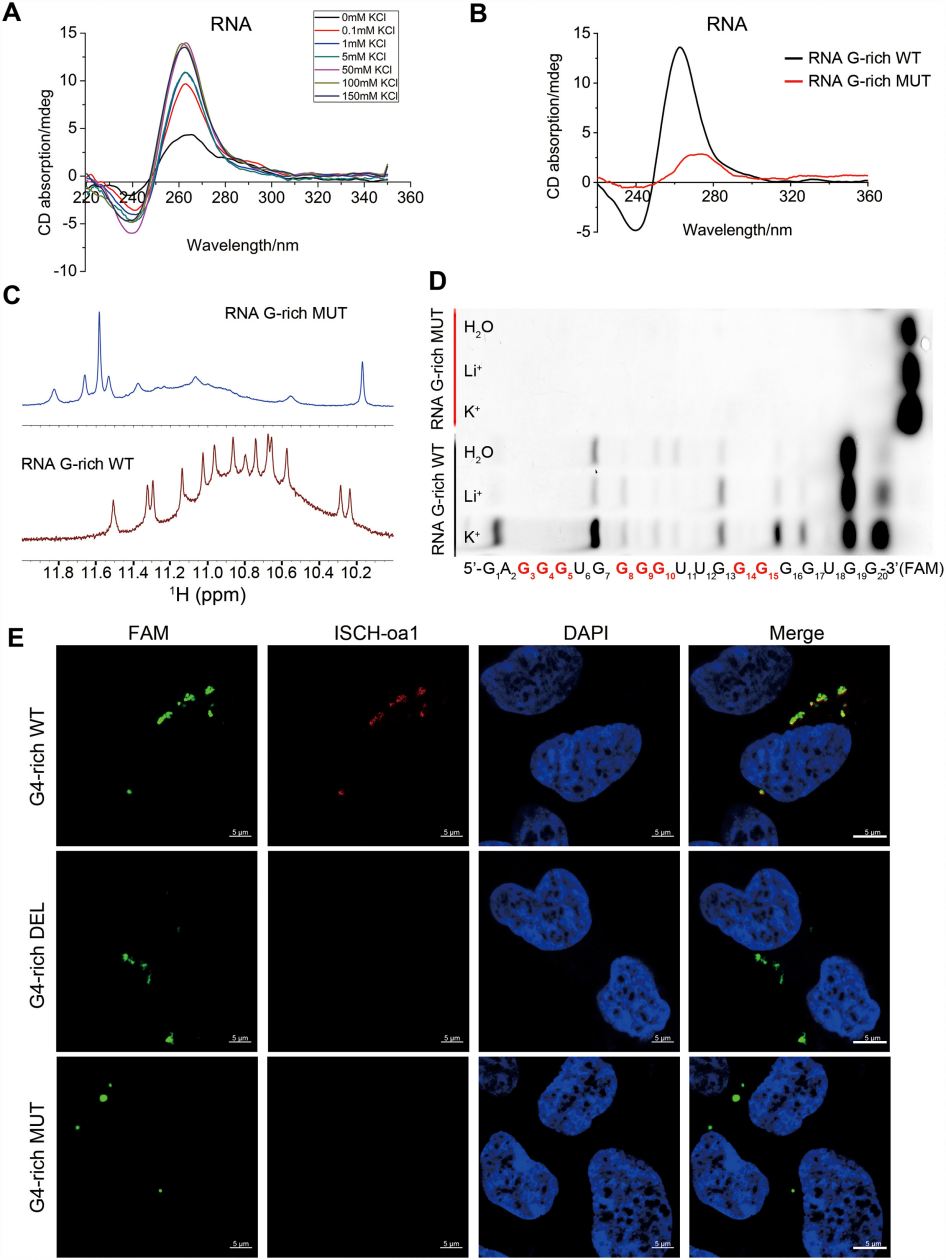
Formation of the intragenic rG4 in pri-miR-23b/27b/24-1. (**A**) CD absorptivity shows characteristic K^+^-dependent spectra of rG4. (**B**) CD absorptivity identified the rG4 characteristic peaks in WT RNA G-rich sequence, which was abolished in the mutated G-rich sequence. (**C**) ^1^H NMR spectrum of the rG4 shows 14 characteristic peaks in the WT RNA G-rich sequence, which disappeared in the mutated G-rich sequence. (**D**) The RNase T1 protection assay of rG4. Eight crucial guanines involved in the rG4 formation are marked in red. The bands for the WT sequence showed the protection of guanines from T1 RNase cleavage. The mutated G-rich sequence without an rG4 structure resulted in all of the guanines except the 3′-terminal guanine were distinctly cleaved in water, Li^+^ or K^+^ solution, showing as one band in probing gel. (**E**) Visualization of rG4 in the cells transfected with WT, deleted, or mutated RNA G-rich sequence. Green: FAM-labeled RNA oligonucleotide; red: rG4 probe; Blue: nucleus; scale bar, 5 μm.

RNA G-quadruplexes (rG4s) are gaining increasing attention, and show higher stability than DNA G4 (23). RNA G4s were recently identified by rG4 sequencing (24) to exist widely in human cells (25), and are emerging as important non-canonical structures involved in translational and post-transcriptional gene regulation (26). Based on the above mentioned finding, we next explored whether the G-rich sequence in pri-miR-23b/27b/24-1 forms rG4. First, G4RNA screener (https://omictools.com/g4rna-screener) (27) was used to predict the formation of an RNA G4 by the aforementioned G-rich sequence, and the data showed the highest scores in the primary transcript of this miR-cluster (pri-miR) (Figure 1A, GAGGGUGGGGUUGGGGGUGG, 1.0 G4NN score, 2.5 G4Hunter score and 700 cGcC score). This suggested that the RNA G-rich sequence had great potential to form rG4. To evaluate whether the G-rich RNA sequence can form G-quadruplexes, we performed CD, ESI-MS, NMR and DMS footprinting experiments. As shown in Figure 2A, CD spectroscopy revealed that the G-rich RNA sequence showed K^+^-dependent formation of a G4 structure, indicating that it formed parallel G-quadruplexes. The mutated G-rich sequence lost the characteristic absorption peak for the G4 (Figure 2B). The CD spectra revealed that the human RNA G-rich sequence also formed an rG4 structure (Supplementary Figure S3C). Furthermore, the ^1^H NMR spectrum of the RNA G-rich sequences in 100 mmol/l KCl solution displayed 14 peaks of partially resolved imino protons at δ10.0–12.0 ppm, which disappeared in the G-rich mutation sequence (Figure 2C). Since the three-layered G-quadruplex consists of 12 guanosines participating in G-tetrads, the 14 imino proton peaks suggested that there might be multiple G-quadruplex topologies formed by the RNA G-rich sequence. This was also supported by the RNase T1 footprinting results, as shown in Figure 2D. Eight guanosines (G3–5, G8–10, G14–15) were completely protected against the T1 RNase cleavage under K^+^ conditions, indicating their crucial role in the multiple rG4 structure. However, some guanosines in the WT G-rich sequence showed protection from T1 RNase cleavage even in water and Li^+^ solution, suggesting its tendency to form complex topologies. When the guanines participating in the G-tetrad were mutated to uracils to destroy the G4 structure even in the K^+^ solution and resulted in all the guanines except the 3′-terminal guanine were distinctly cleaved by T1 RNase. Besides, mutations within these different crucial guanosines abolished the G-quadruplex structure as shown in the NMR and RNase T1 footprinting results (Supplementary Figure S7). These data suggested that the G-rich sequence within pri-miR-23b/27b/24-1 formed a parallel three-layered rG4 with multiple topologies.

To visualize the rG4 structure formed by the G-rich sequence within the miR-23b/27b/24-1, RNA G-rich WT, DEL and MUT sequence labelled with 6-FAM were transfected into HEK293A cells. After hybridization with the rG4 probe-anti-G4 tail sequence (Supplementary Figure S1), the cells transfected with RNA G-rich WT sequences exhibited strong fluorescent spots in the cytoplasm, but not in cells with G-rich DEL or MUT sequences (Figure 2E and Supplementary Figure S8). In the parallel control assay, the hybridization with anti-G4 tail sequence labelled with Cy5 (Supplementary Figure S1) was used as the positive reference, and fluorescent spots were observed in the cells transfected with RNA G-rich WT, DEL and MUT sequences (Supplementary Figure S9). These results demonstrated that the G-rich sequence within miR-23b/27b/24-1 could form specific rG4 structures.

**Figure 3.**
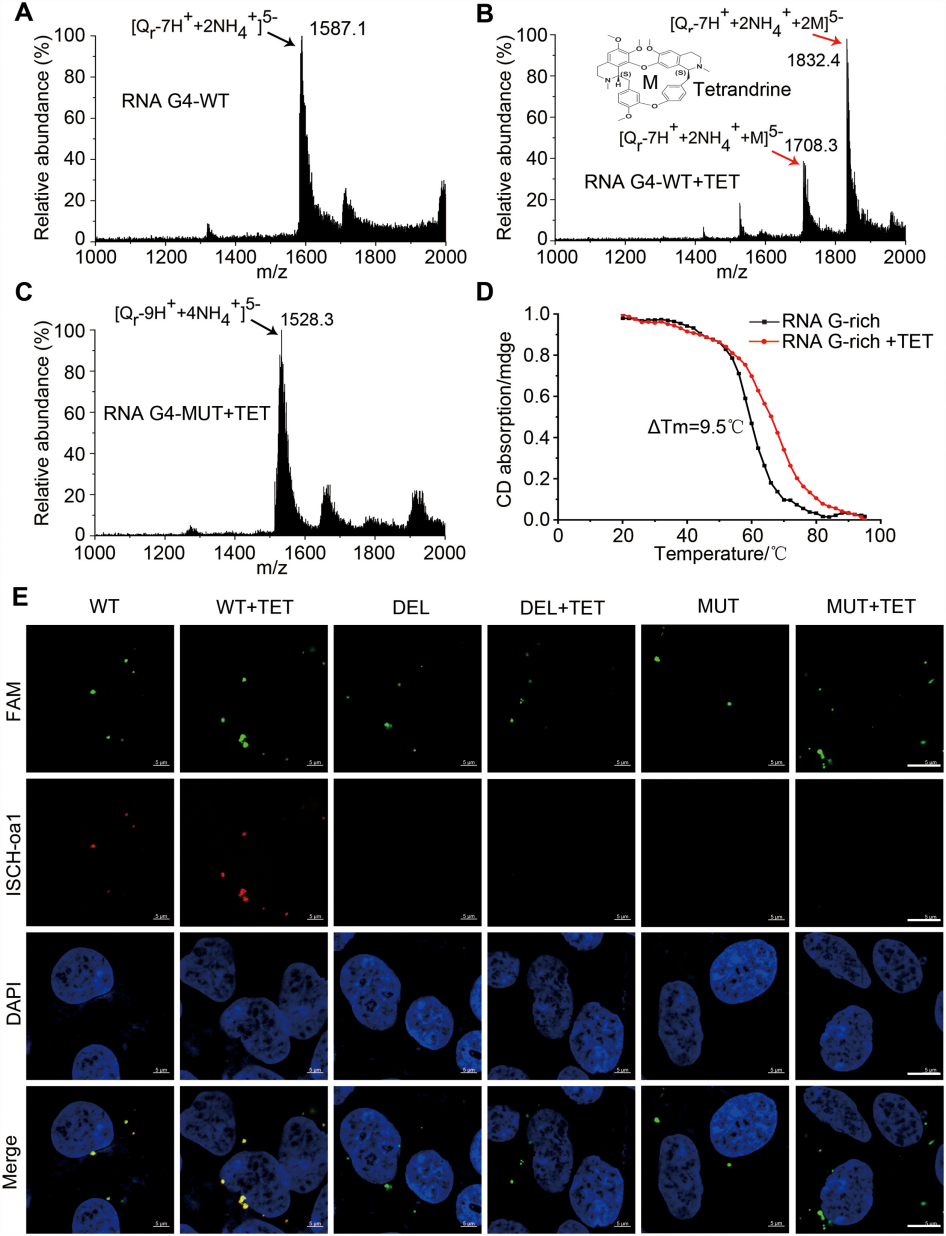
The intragenic rG4 in pri-miR-23b/27b/24-1 was stabilized by TET. (**A**) ESI mass spectrum of RNA G-rich sequence. (**B**) ESI mass spectrum of RNA G-rich sequence with TET. (**C**) ESI mass spectrum of RNA G-rich mutation sequence with TET. (**D**) CD was performed at variable temperatures on the rG4 sequence with/without TET. (**E**) Visualization of rG4 in the cells transfected with WT, deleted, or mutated RNA G-rich sequence with/without TET. Green: FAM-labeled RNA oligonucleotide; red: rG4 probe; Blue: nucleus; scale bar, 5 μm.

Moreover, ESI-MS showed a peak representing the complex ion [Q_r_−7H^+^+2NH_4_^+^]^5−^ at *m*/*z* = 1587.1, which suggested the RNA G-quadruplex contains three G-tetrad layers as it is well known that the ammonium ions locate between two adjacent G-tetrads (Figure 3A). In the presence of TET, a small molecule G4-ligand derived from natural herb(5), the RNA G-rich sequence showed two peaks ([Q_r_−7H^+^+2NH_4_^+^+M]^5−^ at *m*/*z* = 1708.3 and [Q_r_−7H^+^+2NH_4_^+^+2M]^5−^ at *m*/*z* = 1832.4) (Figure 3B), indicating that one or two TET molecules are able to bind the rG4. Conversely, ESI-MS spectra of the mutated rG4 sequence showed no TET binding activity (Figure 3C). Furthermore, CD measurements with variable temperature revealed increased Tm of the rG4 upon exposure to TET (Figure 3D), suggesting that TET stabilized the rG4. Meanwhile, fluorescence in the cells transfected with the RNA G-rich WT sequence was greatly enhanced by TET treatment (10^−6^ mol/l), but not in cells transfected with RNA G-rich DEL or MUT sequence (Figure 3E and Supplementary Figure S10). This result was consistent with the *in vitro* ESI-MS and CD studies, showing that TET exhibits considerable selectivity for the rG4 formed by the G-rich sequence within the miR-23b/27b/24-1. Based on the above results, we speculated that TET down-regulation of pri-miR-23b/27b/24-1 expression was dependent on the stabilization of intragenic rG4 sequences.

### The rG4 suppresses Drosha-DGCR8-binding and processing of pri-miR-23b/27b/24-1

**Figure 4.**
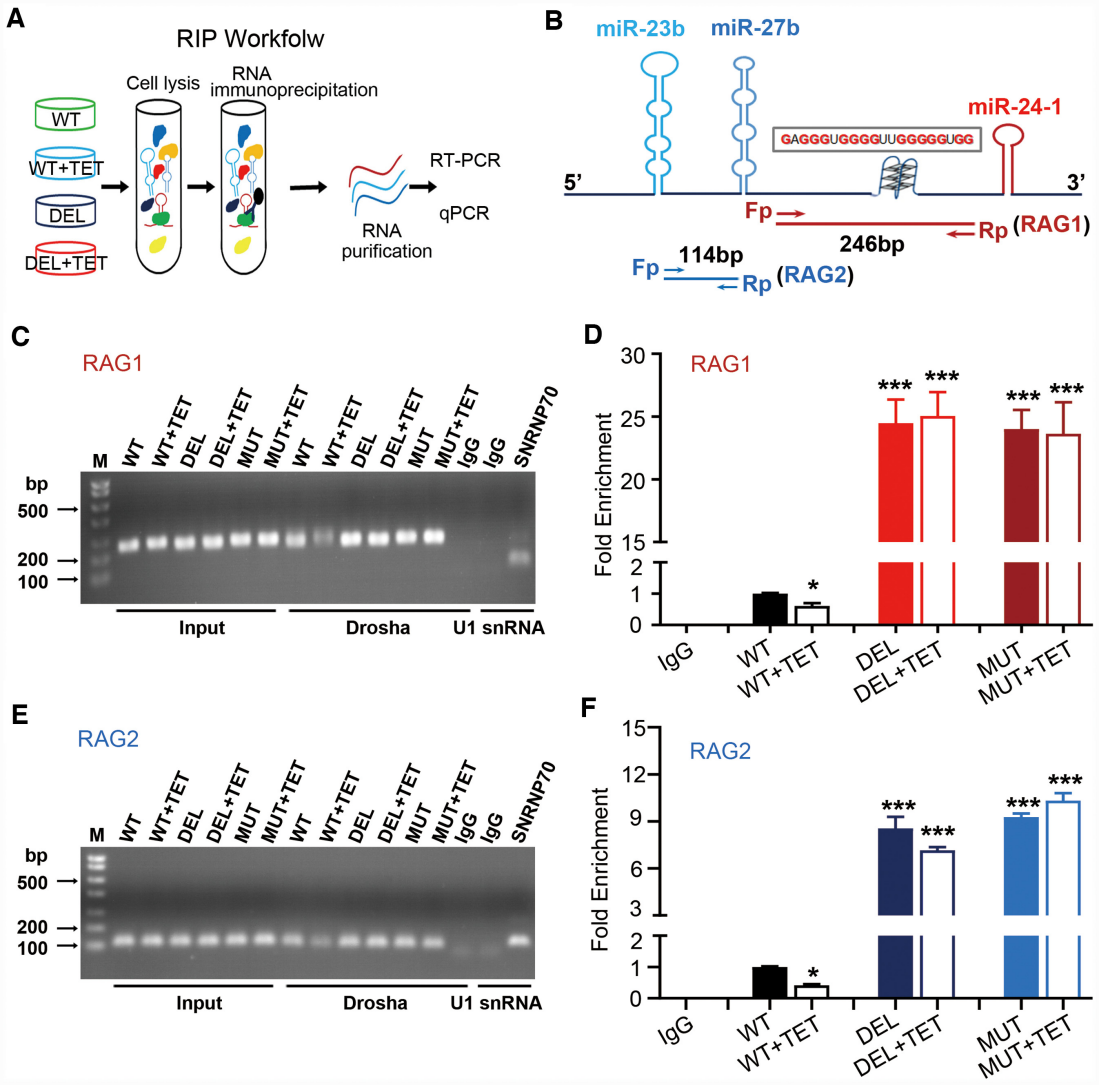
RNA G4 inhibited Drosha to edit the pri-miRs cluster, leading to reduced mature miRs expression. (**A**) Scheme showing the workflow of RNA immunoprecipitation (RIP). (**B**) The location of PCR products using specific primers for RIP. (C and D) Semi-quantitative PCR (**C**) or real-time PCR (**D**) analysis of the RAG1 (interval between pri-miR-27b∼pri-miR-24-1, including the G-rich sequence) enriched by Drosha in HEK293A cells transduced with WT-ADV, DEL-ADV and MUT-ADV for 24 h and treated with TET (10^−6^ mol/l) for 12 h. (E and F) Semi-quantitative PCR (**E**) or real-time PCR (**F**) analysis of the RAG2 (interval between pri-miR-23b∼pri-miR-27b) enriched by Drosha in HEK293A cells transduced with WT-ADV, DEL-ADV and MUT-ADV for 24 h and treated with TET (10^−6^ mol/l) for 12 h. RIP using IgG and anti-SNRNP70 as negative and positive controls, respectively. Graphs shown are representative of three independent experiments, and the data are shown as mean ± SEM from three independent experiments (*P < 0.05; ***P < 0.001).

To determine how the rG4 elicits its negative regulation on miR biogenesis, we next questioned whether the rG4 may prevent the interaction of pri-miR-23b/27b/24-1 with Drosha, the key initiative component of the canonical pri-miR microprocessor (28,29). An RNA immunoprecipitation (RIP) assay was performed with anti-Drosha using HEK293A cells transduced with WT-ADV or DEL-ADV, each with TET or mock treatment (Figure 4A). RNAs from the input and RIP were analyzed by semi-quantitative RT-PCR and RT-qPCR using two sets of specific primers in the pri-miR-23b/27b/24-1 cluster located between the pre-miR stem loops as illustrated in Figure 4B. In parallel RIP experiments, IgG was used as a negative control and anti-SNRNP70 as a specificity control (Supplementary Figure S11). As shown in Figure 4, the semi-quantitative RT-PCR (Figure 4C) and real-time PCR (Figure 4D) product of RNA fragment (RAG1, including the G-rich sequence) binding with Drosha decreased when HEK-293A cells transfected with WT-ADV were treated with TET (10^−6^ mol/l). Importantly, deletion and mutation of the G4 markedly enhanced the association of Drosha with the pri-miRs, which failed to respond to TET-treatment. (Figure 4C and D). Moreover, we identified the sequences between pri-miR-23b and pri-miR-27b (RAG2) from RIP, using semi-quantitative RT-PCR and real-time PCR. The results were consistent with the data of RAG1 sequence containing the G-rich sequence (Figure 4E and F). This demonstrated that the rG4 inhibited Drosha binding and cleavage of the pri-miRs cluster.

The Drosha-DGCR8 complex is required for the cleavage of pri-miRNA (30,31). To further illustrate the effect of rG4 on Drosha's cleavage of the pri-miRNA, the parallel RIP experiments with DGCR8 were performed. As shown in Supplementary Figure S12, the RAG1 fragment binding with DGCR8 decreased when cells transfected with WT-ADV were treated with TET (10^−6^ mol/l), and the deleted and mutated G4-rich sequences pulled down by anti-DGCR8 antibody increased compared to that in WT. The results of RAG2 sequence were consistent with those of RAG1. Taken together, these results showed that the rG4 had an adverse effect on the cleavage processing of pri-miRNAs by the Drosha-DGCR8 complex.

### Disruption of the G4 in the rat genome by CRISPR/Cas9 aberrantly increased miR-24, miR-27b and miR-23b production in the heart, resulting in cardiac contractile dysfunction

**Figure 5.**
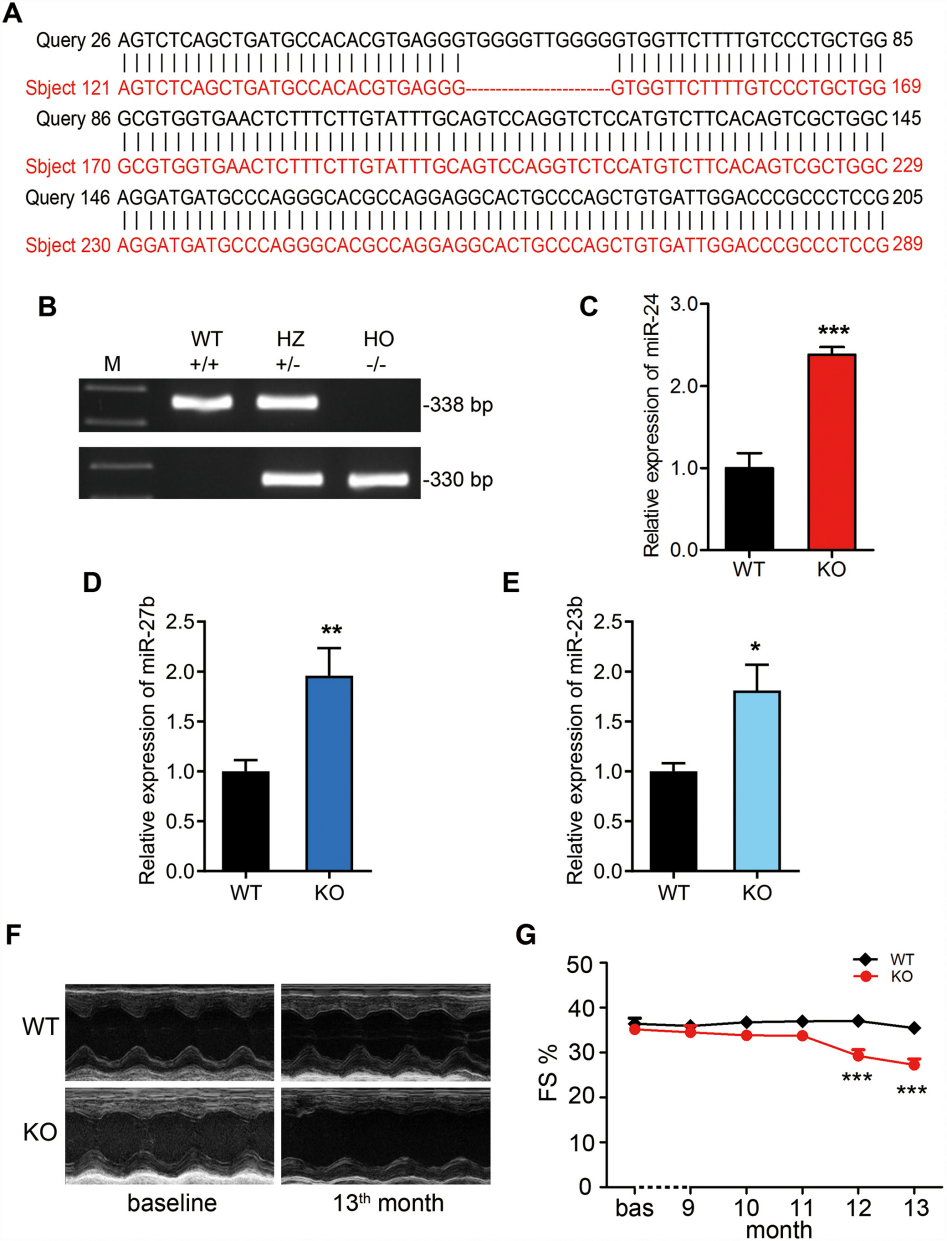
Deletion of the G4 led to higher expression of miR-24, miR-27b, and miR-23b, and cardiac contractile dysfunction in rats. (**A**) Illustration of deleted sequences in homozygous G4-KO rats as compared to the wild type sequence based on Blast (https://blast.ncbi.nlm.nih.gov/Blast.cgi). (**B**) Genomic DNAs were analyzed with PCR in wild type (WT), heterozygous (HZ) and homozygous (HO) rats. (C–E) RT-qPCR for detecting miR-24 (**C**), miR-27b (**D**) and miR-23b (**E**) expression in cardiac tissues of WT and G4-KO rats. (**F**) Representative M-mode echocardiography in WT and G4-KO rats. (**G**) Echocardiography revealed a decrease in fractional shortening (FS %) in G4-KO rats as compared to that in the WT rats (*P< 0.05, **P< 0.01, ***P< 0.001, *n* = 7).

To investigate the biological functions of the G4 in governing miR-23b, miR-27b and miR-24 expression *in vivo*, we used CRISPR/Cas9 to generate a rat line in which the G4 forming sequence was partially deleted (G4-KO) (Supplementary Figure S13A). Whole-genome sequencing identified a number of rat lines that harbor various deletions in the G4 (Supplementary Figure S13B) without off-target mutations (Supplementary Figure S13C). We selected the homozygous rat lines #3–6, in which the sequence (5′-TGGGGTTGGGG-3′) was successfully deleted (Figure 5A and Supplementary Figure S13B), for further analysis. Homozygous G4-KO rats were identified by PCR genotyping (Figure 5B). Importantly, miR-24, miR-27b and miR-23b were all increased in the G4-KO rat cardiac tissue compared with that in WT (Figure 5C–E). Similar increase in miR-24, miR-27b and miR-23b were also detected in the NRCMs and neonatal rat cardiac fibroblasts (NRCFs) of the G4-KO rats (Supplementary Figure S14). These results demonstrated that the G4 in the miR-23b/27b/24-1 cluster suppressed the expression of endogenous miR-23b, miR-27b and miR-24 *in vivo*. To determine whether disruption of the G4 affects cardiac contractile function *in vivo*, M-mode echocardiography was performed in G4-KO rats and WT rats (Figure 5F). The fractional shortening percentage (FS %) in G4-KO rats was significantly reduced at the 12th and the 13th month of age (Figure 5G). This suggested that disruption of the G4 aberrantly increased production of the three miRNAs in the heart, thereby decreasing cardiac contractile function in rats.

To investigate the potential mechanism by which G4 affects cardiac function though three miRNAs down-regulation, miRanda (http://www.microrna.org/microrna/home.do), miRDB (http://mirdb.org) and psRobot (http://omicslab.genetics.ac.cn/psRobot) were combined used to search for the downstream targets of the miR-23b/27b/24-1 cluster, identifying putative mRNA targets for miR-23b (*n* = 22), miR-27b (*n* = 75), and miR-24 (*n* = 95) (Supplementary Figure S15A). The Kyoto Encyclopedia of Genes and Genomes (KEGG) pathway analysis showed that many pathways, including the calcium signaling pathway, that play an important role in cardiac contraction (*P*< 0.05, Supplementary Figure S15B). Based on these results and a literature search, we selected the putative targets of miR-24, miR-27b and miR-23b involved in calcium signaling. These targets have been reported to bind with miR-24, miR-27b and miR-23b. Among these predicted mRNA targets, our previous study and another study have demonstrated that the 3′ untranslated regions (3′UTRs) of JP2, EHD3, and SMAD5 mRNA harbored miR-24, miR-27b and miR-23b binding sites, respectively (32,33). These siRNA target sites are conserved in rats and humans (Supplementary Figure S15C-E). Functionally, JP2 is essential for effective excitation–contraction (E–C) coupling in cardiomyocytes (32). EHD3 is a key regulator of atrial myocyte excitability and cardiac conduction (34,35). SMAD5 plays important roles in regulating cardiac contractile through changes in calcium-handling proteins and myofilament proteins (33,36). Therefore, dysregulation of these genes will result in serious cardiac diseases. Importantly, the aberrant increase in miR-24, miR-27b and miR-23b, due to the loss of the intragenic G4, resulted in significantly reduced expression of the aforementioned target genes at both mRNA and protein levels (Supplementary Figure S16A-F). Taken together, these results suggested that loss of the conserved G4 results in reduced expression of multiple key genes that govern the E–C coupling efficiency and calcium signaling transduction of cardiomyocytes through the collective effects of distinct miRs encoded by the miR-23b/27b/24-1 cluster.

### Loss of the G4 affected LCC-RyR coupling efficiency of cardiomyocytes and caused cardiac contractile dysfunction

**Figure 6.**
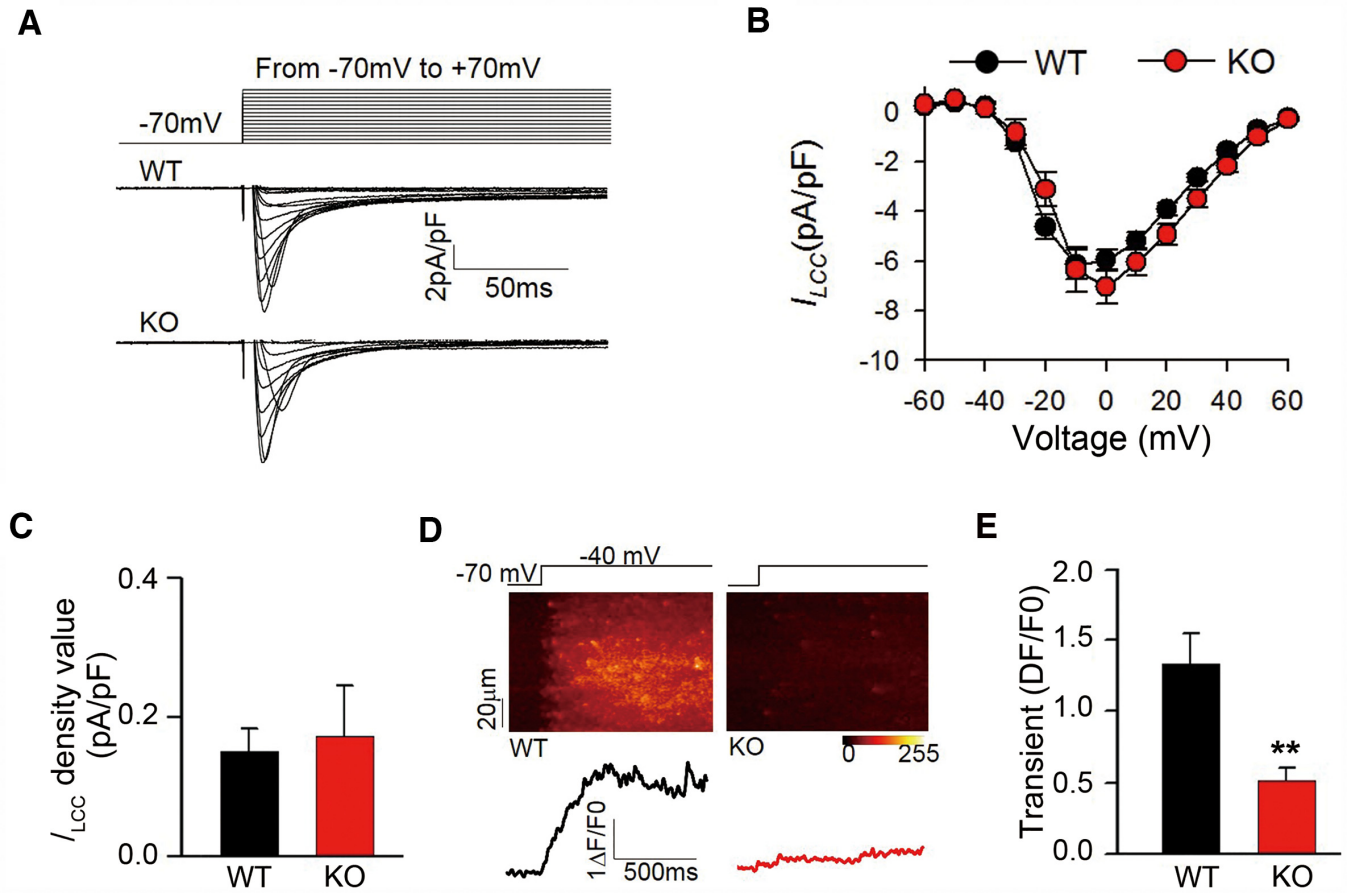
Deletion of the G4 leads to decreased LCC-RyR coupling in cardiomyocytes. (**A**) Calcium currents triggered by depolarization from –70 mV to different voltages (–70 to +70 mV) in the cardiomyocytes of WT and G4-KO rats. (**B**) The LCC current density in the cardiomyocytes of WT and G4-KO rats. (**C**) The LCC current density at -40 mV determined by ILCC = *G* (*V*− *VR*), where *G* is the LCC conductance, *V* is the membrane potential and VR is the reverse potential. (**D**) Representative images showed that depolarization from –70 to –40 mV activated calcium release by the RyR. (**E**) The calcium transient at –40 mV in the cardiomyocytes of WT and G4-KO rats. Representative images of seven mice per group are shown (**P< 0.01)

Based on the loss of G4 leading to up-regulation of miRNAs and down-regulation of their target genes involved in E–C coupling, we further explore the effect of G4 on cardiomyocytes Ca^2+^ signaling. The contraction of myocardium is known to be determined by the excitation-contraction coupling process, where the Ca^2+^ influx through LCCs (ILCC) activates Ca^2+^ release from RyRs (37,38). Combining patch clamp analyses and confocal imaging, whole-cell Ca^2+^ signals were measured in the isolated mature cardiomyocytes from G4-KO and WT rats. The whole-cell patch clamp (Figure 6A) experiment showed that neither ILCC density nor its voltage dependence differed between the WT and G4-KO cardiomyocytes (Figure 6B). Therefore, we utilized a recently established method (39) to detect the change in LCC-RyR signaling efficiency in cardiomyocytes loaded with the Ca^2+^ indicator Fluo-4. Upon depolarization from –70 to –40 mV, the ILCC density was comparable in both groups (Figure 6C). Such a suprathreshold ILCC triggered a moderate transient of RyR Ca^2+^ release in the WT cardiomyocytes but only sparse Ca^2+^ sparks in those of G4-KO (Figure 6D). The average strength of RyR Ca^2+^ release was significantly reduced in G4-KO cardiomyocytes (Figure 6E). These results indicate defects in the LCC-RyR coupling in G4-KO cardiomyocytes. The profoundly decreased efficiency of LCC-RyR signaling suggested that the G4-dependent suppression of the miR-23b/27b/24-1 miR pathway is essential for normal cardiac contractile function by maintaining E–C coupling in cardiomyocytes.

## DISCUSSION

**Figure 7.**
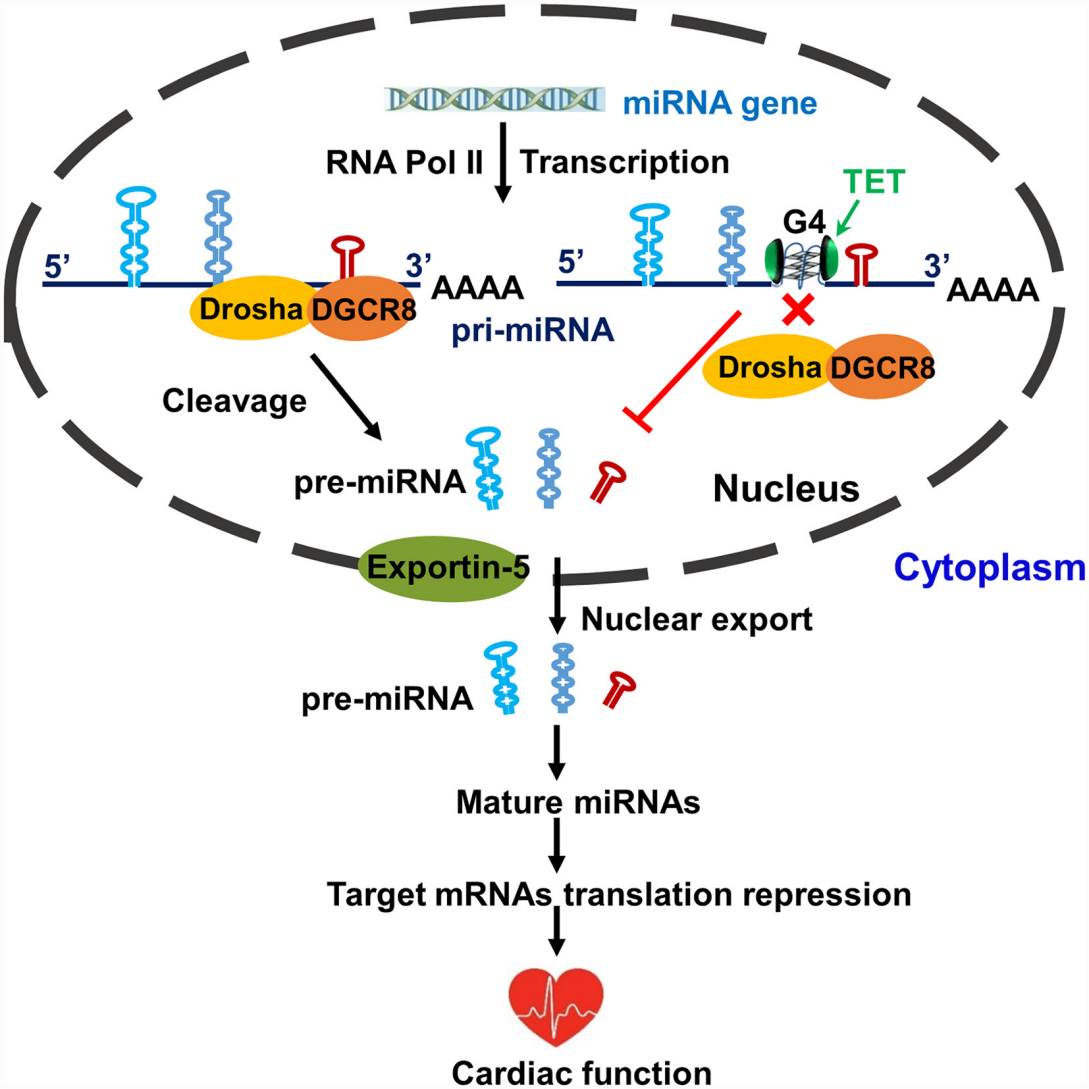
Schematic diagram depicting the important role of G4 in this study. We found that the formation of an intragenic RNA G4 located within the miR-23b/27b/24-1 cluster inhibited Drosha-DGCR8 cleavage of pri-miRNAs. Moreover, using CRISPR/Cas9 to knockout G4 will increase three miRNAs expression and reduce their downstream targets, leading to electrical remodeling and cardiac dysfunction.

In this study, we performed a series of biophysical and pharmacological experiments to demonstrate the formation of a conserved intragenic rG4 located within the miR-23b/27b/24-1 cluster. Further investigation indicated that this rG4 inhibited the Drosha-DGCR8 association and cleavage of the pri-miRs, thus suppressing the biogenesis of mature miRs. Moreover, using the advanced genome editing technology of CRISPR/Cas9, our studies provided the first evidence for the biological importance of this G4 in maintaining normal heart function through governing miR biogenesis and regulation of the downstream targets. Based on these findings, a comprehensive model for this novel G4–miR pathway in cardiac function is illustrated in Figure 7.

The involvement of G4s in numerous biological processes and the pathogenesis of various diseases has been increasingly recognized (40,41). In addition to their important roles in telomere extension and DNA replication, many G4s are implicated in regulation of protein coding genes in *cis*, in which DNA G4s are located at the promoter regions to regulate transcription whereas rG4s located in untranslated regions regulate mRNA processing and translation (42,43). However, a prevailing knowledge gap exists for *in vivo* evidence for rG4 function and the biological consequences of rG4 disruption. There are some controversial theories about rG4 existence, according to which many rG4s sequences in eukaryotic and human cells can fold into rG4 structures *in vitro*, but actively unfold *in vivo* possibly because of specific factors and robust and effective machinery (44). However, a recent study demonstrated that rG4 showed higher stability than DNA G4 (23), suggesting the possibility of rG4 existence *in vivo*. An rG4-specific fluorescent probe was used to detect rG4 structures in the living HeLa cells (45). Ivanov *et al.* rationalized the role of naturally occurring rG4-assembling tRNA fragments in the regulation of mRNA translation (43). In the present study, we demonstrated that the rG4 in the miR-23b/27b/24-1 cluster decreased the levels of three miRs, which was consistent with a recent report suggesting the involvement of rG4s in the processing of pri-miRs in cultured cells (46). More importantly, our study provided the first evidence of G4 function *in vivo* by demonstrating that the loss of G4 in the miR cluster causes defects in cardiac systolic function.

Notably, aberrant increase in miRs encoded by the miR-23b/27b/24-1 cluster is indicated in multiple aspects of cardiac malfunction in recent studies, suggesting that co-dysregulation of these miRs may trigger and/or advance cardiomyocyte malfunction (32,47,48). Our bioinformatic analysis showed that many target genes of miR-23b/27b/24-1 were involved in calcium signaling pathways, suggesting that the G4-governed miR-23b/27b/24-1 biogenesis plays important roles in maintaining normal calcium signaling in cardiomyocytes and cardiac contraction. Our previous studies also revealed that elevated miR-24 caused JP2 reduction, which consequently leads to defects in LCC-RyR coupling and contractile function in failing heart cells (32). In the present study, we further showed that expression of the miR-24-JP2 pathway is regulated by this G4. Besides the miR-24-JP2 pathway, the G4-governed expression of EHD3, a target gene of miR-27b and an important regulator of atrial myocardial excitation, could also contribute to the excitation contraction coupling efficiency. Additionally, the G4-controlled miR-23b target SMAD5 is an important determinant of the TGF-β signaling pathway, which plays critical roles in electrical remodeling including E-C coupling and intracellular Ca^2+^ transduction (49,50). Abnormal expression of miR-27b and miR-23b also induces cardiac hypertrophy and decreases fractional shortening. Thus, defects in the G4-regulated 23b/27b/24-1 pathway may contribute to various steps toward heart failure (47,51).

Although both DNA and RNA G4s can form, the regulation of mature miR abundance in our study is most likely through post-transcriptional processing of the pri-miR, because TET-mediated stabilization of the rG4 reduced mature miR production without affecting the miR-23b/27b/24-1 cluster pri-miR. Encouraged by these results, we speculated that rG4 had an adverse effect on the processing of pri-miRNAs via the enzyme Drosha (28). Our subsequent experimental data further indicated that the G4 acts to prevent Drosha binding and cleavage of the 23b/27b/24-1 cluster pri-miR, thus simultaneously inhibiting the biogenesis of all the miRs encoded by the 23b/27b/24-1 cluster (Figure 4). In addition to Drosha, DGCR8 is also required to mediate the cleavage of pri-miRNA (31,52). Consistent with the finding that rG4 affects Drosha's binding to pri-miR-23b/27b/24-1, rG4 interfered with the binding of DGCR8 to pri-miRNA. Although these remarkable results were observed here, we could not assign a definitive function of the rG4 directly to DGCR8 within the pri-miRNA cluster. This suggests that DGCR8 could help to orient the specific positioning of the Drosha cleavage site in the pri-miRNA (52). Whether the rG4 affects the positioning of DGCR8 in pri-miRNAs, leading to the inhibition of Drosha cleavage, needs to be confirmed in future studies. We cannot rule out the possibility that the rG4 influenced Drosha binding to pri-miRNA indirectly affects the binding of DGCR8 with pri-miRNA. Perreault *et al.* proposed that the position of rG4 relative to the Drosha cleavage site possibly dictated the effect of rG4 on pri-miRNA processing, in which the loss of rG4 decreased miR-200c and miR-497 but increased miR-451a levels (46). The current available experimental data were not relatively sufficient to permit us to assign the effect of G4 distance on miRNA biogenesis. Interestingly, bioinformatic analysis was performed to determine the similar rG4s located in or close to miRNAs cluster reported by the previous studies (53). We found that many similar rG4s were close to miRNAs clusters (miR-200b/200a/429, mi-29c/29b-2, miR-368/376a, miR-212/132, miR-99b/let7e/125a, miR-192/194–2, miR-200c/141 and miR-144/451a) in human and rat genomes. Moreover, there were multiple G-rich sequences in the regions between two miRNAs, or close to one of the two miRNA precursors simultaneously with high RNA G4 scores, calculated with G4RNA screener in some miRNA clusters. These multiple rG4s may regulate the biogenesis of miRNA clusters simultaneously. As for whether there is a potential regularity in which the distance of rG4 to the pri-miRNA cluster down-regulates or up-regulates miR cluster biogenesis is a worthy question to explore in future studies.

In summary, our study unravels a novel mechanism of rG4-mediated miRs cluster via inhibition of the Drosha-DGCR8 association and cleavage of the pri-miRs. Although the precise molecular network downstream of the G-4-controlled miR cluster in the cardiovascular system remains elusive, our findings demonstrated a novel mechanism for efficient control of heart function through this highly organized regulatory molecular hub by co-regulating distinct miRs. Moreover, the discovery of the role of TET in modulating the rG4 stability offers potential pharmacological strategies against cardiac malfunction caused by dysregulation of the miR-23b/27b/24-1 pathway.

## Supplementary Material

gkab055_Supplemental_File
