# Supplementary material for: Novel roles of an intragenic G-quadruplex in controlling microRNA expression and cardiac function
Source: Nucleic Acids Res. 2021 Feb 9;49(5):2522–36. doi: 10.1093/nar/gkab055 (PMC7969000; doi:10.1093/nar/gkab055)
Supplement: gkab055_Supplemental_File [file gkab055_supplemental_file.pdf]

SUPPLEMENTAL INFORMATION

SUPPLEMENTAL EXPERIMENTAL PROCEDURES

DMS Footprinting

The labeled DNA G-rich sequence (Supplementary table S2) was diluted with 60 mmol/L Tris-HCl buffer (pH 7.4) to 0.2 μmol/L while containing 150 mmol/L salt (LiCl or KCl). Afterwards, pre-annealing at 95 ℃ was performed for 10 min before slowly cooling to 4 ℃. The annealed samples were treated with 10% DMS for 5 min before being quenched and extracted with a Tris-phenol-chloroform solution (pH 8.0). The aqueous phase was precipitated using ethanol at -80 ℃. The dry precipitate was dissolved in 10% piperidine and incubated at 90 ℃ for 30 min, followed by precipitation. The treated sample was resolved using 20% denatured PAGE gel at 1500 V for 4 hours in 4 ℃ (1).

Generation of G-rich sequence upstream of miR-24-1 knockout rats

G-rich sequence upstream of miR-24-1 knockout rats were constructed by CRISPR/Cas9 technique (2), online CRISPRR design tool (http://crisprr.mit.edu) was used to predict the guide sequences of the target site. A pair of oligomer (oligo1: 5'-TAGGCGTGAGGGTGGGGTTGGGGG-3') and oligo2: 5'-AAACCCCCCAACCCCACCCTCACG-3') was annealed and cloned into the restriction site of pUC57-sgRNA (Add gene 51132). The DNA was amplified by PCR used primers spanning the T7 promoter and sgRNA sequences (Forward primer: 5'-GATCCCTAATACGACTCACTATAG-3', Reverse primer: 5'-AAAAAAAGCACCGACTCGGT-3'). Then, sgRNA was transcribed and purified using the MEGAshortscript Kit (Amibion, AM1354) and miRNeasy Micro Kit (QiaGen, 217084), respectively. Cas9 expression vector (Addgene 44758) was linearized with PmeI and in vitro as the template, transcribed using the T7 Ultra Kit (Ambion, AM1345). The mRNA was purified using the RNeasy Mini Kit (QiaGen, 74104). Cas9 and sgRNA mRNA were injected to one-cell embryos by FemtoJet 5247 microinjection system. Rat tail genomic DNA was extracted by phenol-chloroform and alcohol precipitation to identify the F0 offspring. Mutant allele in 4 founder rats were sequenced by chromatogram, which revealed different in deletion mutations in the CRISPR/Cas9 target site, and founder #3-6 with an 11-bp deletion was used to generate homozygous rats with loss of the G-rich sequence.

Identification of G-rich sequence upstream of miR-24-1 knockout rats

Rat tail genomic DNA was extracted by phenol-chloroform and alcohol precipitation, PCR analysis was performed with the primers: primer C (5'-TACCACTGCTGTTGGGTCTG-3') and W (5'-CACGTGAGGGTGGGGTTG-3') or a pair of C and K (5'-ACACGTGAGGGGTGGTTC-3'). The homozygous rats showed one band at 330bp, the heterozygous rats showed two bands at 338bp and 330bp, and the wild type rats showed one band at 338bp. The PCR product of homozygous rats was sequenced and run blast in NCBI (https://blast.ncbi.nlm.nih.gov/Blast.cgi). In order to identify the effects deletion of G-rich sequence upstream of miR-24 on other genes, whole-genome sequencing (3) services were provided by BGI.

Protein extracts and western blotting

Proteins from the rat heart NRCMs and HEK-293A cell were extracted in buffer containing 1% sodium deoxycholate, 10 mmol/L $Na_4P_2O_7$, 1% Triton X-100, 10% glycerol, 150 mmol/L NaCl, 5 mmol/L EDTA $\cdot Na_2$, 50 mmol/L Tris (pH 7.4), 0.1% SDS, 50 mmol/L NaF, 1 mmol/L Na_3VO_4, 1 mmol/L PMSF, and protease inhibitor cocktail (Roche). Separated proteins by SDS-PAGE were transferred to Nitrocellulose (NC) membranes. Membranes were incubated with primary antibodies and then probed with horseradish peroxidase- conjugated secondary antibodies. Blots were visualized with the use of an enhanced chemiluminescence kit (Amersham Bioscieces lnc., Piscataway, NJ, USA). Primary antibodies used for western blotting in this study are JP2 (Aviva Systems Biology), EHD3 (Proteintech Group), SMAD5 (Proteintech Group) and GADPH (Cell Signaling Technology).

Immunofluorescence staining

The Immunofluorescence staining for stem cells and human-induced pluripotent stem cell-derived cardiomyocytes was performed as described previously(4). The cells were plated on 20 mm coverslips and were fixed with 4% paraformaldehyde for 10 minutes, followed by washing with PBS three times for 10 minutes. And the cells were treated with 0.5% Triton X-100 for 30 minutes, and treated with 3% bovine serum albumin (BSA) at room temperature for 1 hour. Cells were incubated with primary antibodies including NANOG (1:100, Abcam), TRA-1-60 (1:100, Santa Cruz), TNNT2 (1:100, Santa Cruz), α-actinin (1:100, abcam) for overnight at 4 ℃, followed by 1:200 Alexa Fluor secondary antibodies (Life Technology). After the cells were washed, they were studied using a confocal microscope (Carl Zeiss, LSM-510).

REFERENCES

1. Chen, H., Long, H., Cui, X., Zhou, J., Xu, M. and Yuan, G. (2014) Exploring the formation and recognition of an important G-quadruplex in a HIF1alpha promoter and its transcriptional inhibition by a benzo[c]phenanthridine derivative. *J Am Chem Soc*, **136**, 2583-2591.
2. Suzuki, K., Tsunekawa, Y., Hernandez-Benitez, R., Wu, J., Zhu, J., Kim, E.J., Hatanaka, F., Yamamoto, M., Araoka, T., Li, Z. *et al.* (2016) In vivo genome editing via CRISPR/Cas9 mediated homology-independent targeted integration. *Nature*, **540**, 144-149.
3. Kleinstiver, B.P., Pattanayak, V., Prew, M.S., Tsai, S.Q., Nguyen, N.T., Zheng, Z. and Joung, J.K. (2016) High-fidelity CRISPR-Cas9 nucleases with no detectable genome-wide off-target effects. *Nature*, **529**, 490-495.
4. Cui, N., Wu, F., Lu, W.J., Bai, R., Ke, B., Liu, T., Li, L., Lan, F. and Cui, M. (2019) Doxorubicin-induced cardiotoxicity is maturation dependent due to the shift from topoisomerase IIalpha to IIbeta in human stem cell derived cardiomyocytes. *J Cell Mol Med*, **23**, 4627-4639.

SUPPLEMENTAL FIGURE LEGENDS

Supplemental Figure S1. Detection of RNA G-quadruplexes in cells. (**A**) Scheme of the transfection with FAM-labelled G-rich sequence and the utilization of hybridized probes for rG4 visualization. (**B**) Illustration of G-rich RNA sequences and the rG4 probes (ISCH-oa1) or Cy5 labelled anti-G4 rich tail sequence, which is a 25 bases oligonucleotide complementary to the oligonucleotide sequence adjacent to the 3' end of the G-rich sequence.

Supplemental Figure S2. Formation of DNA G4 in rat genome. (**A** and **B**) CD absorptivity identifies the G4 characteristic peaks in WT DNA, which is abolished in the G-rich sequence deletion (**A**) or mutation (**B**). (**C**) ^{1}H-NMR spectrum of DNA G4. (**D**) DMS footprinting assay of DNA G4.

Supplemental Figure S3. Formation of DNA and RNA G4 in human genome. (**A**) Position of G-rich sequence in miR-23b/miR-27b/miR-24-1 cluster in human genome. (**B**) The CD absorptivity represents DNA G-rich sequence (**C**) The CD absorptivity represents RNA G-rich sequence.

Supplemental Figure S4. Cardiomyocytes are differentiated from human embryonic stem cells (hESCs). Representative immunofluorescence staining of hESCs for markers of pluripotent stem cells, including NANOG (green) and TRA-1-60 (red); scale bar, 25 μm. Immunofluorescent staining for α-actinin (green) and TNNT2 (red) to demonstrate hESCs-CMs; scale bar, 10 μm.

Supplemental Figure S5. Stabilization of the G4 by TET down-regulates miR-24/miR-27b/miR-23b. (**A**) miR-24, miR-27b and miR-23b expression in hESCs-CMs treated with various concentrations of TET for 24 hours (black: miR-24; red: miR-27b; blue: miR-23b). (**B**) miR-24 expression in HEK293A cells transfected with WT-ADV, DEL-ADV, or MUT-ADV for 24 hours and treated with various concentrations of TET for 12 hours (black: WT-ADV; red: DEL-ADV; blue: MUT-ADV).

Supplemental Figure S6. TET didn't affect the miR-27a and miR-23a expression in the NRCMs and hESCs-CMs. (**A**) Schematic diagram of miR-23/27/24 cluster in rat. (**B**) Quantitative PCR analysis of miR-27a and miR-23a expression in the NRCMs treated with TET (10^{-6} mol/L) for 24 hours, respectively. (**C**) Schematic diagram of miR-23/27/24 cluster in human. (**D**) Quantitative PCR analysis of miR-27a and miR-23a expression in the hESCs-CMs treated with TET (10^{-6} mol/L) for 24 hours. The data are shown as mean ± SEM from three independent experiments.

Supplemental Figure S7. The ^{1}H-NMR spectrum and RNase T1 protection assay of rG4. (**A**) ^{1}H-NMR spectrum of rG4 showed 14 characteristic peaks in WT RNA G-rich sequence, which disappeared in the other mutated G-rich sequences. (**B**) The RNase T1 protection assay of WT RNA G-rich sequence and mutated G-rich sequences.

Supplemental Figure S8. Quantification of ISCH-oa1 labelled anti-G4 tail oligonucleotide spots and FAM-labelled G-rich sequences spots in cells. The data shown as mean ± SEM from 100 cells per sample and three independent experiments (***P<0.001).

Supplemental Figure S9. The upper panel shows the visualization of WT or deleted or mutated RNA G-rich sequence transfected cells stained with Cy5-anti-G4 tail sequence. Green: FAM-labeled RNA oligonucleotide; red: Cy5-anti-G4 tail sequence; Blue: nucleus; scale bar, 5 μm. The lower is the quantification of Cy5-anti-G4 tail sequence spots and FAM-labelled G-rich sequences spots in cells. The data shown as mean ± SEM from 100 cells per sample and three independent experiments.

Supplemental Figure S10. Quantification of ISCH-oa1 labelled anti-G4 tail oligonucleotide spots and FAM-labelled G-rich sequences spots in HEK293A cells transfected with WT, DEL, or MUT

G-rich oligonucleotide for 12 hours and treated without or with TET (10^{-6} mol/L) for 12 hours. The data shown as mean ± SEM from 100 cells per sample and three independent experiments (***P*<0.01; ****P*<0.001).

Supplemental Figure S11. The positive control of RIP assay. RIP assay with IgG (negative control) and anti-SNRNP70 (positive control) analyzed by real-time PCR using specific primer U1snRNA. Graphs show mean ± SEM of three independent experiments (****P*<0.001).

Supplemental Figure S12. RNA G4 interfered the binding of DGCR8 to pri-miRNA. (**A**) Real-time PCR analysis of the RAG1 (interval between pri-miR-27b~pri-miR-24-1, including the G-rich sequence) enriched by DGCR8 in HEK293A cells transduced with WT-ADV, DEL-ADV and MUT-ADV for 24 hours and treated with TET (10^{-6} mol/L) for 12 hours. (**B**) Real-time PCR analysis of the RAG2 (interval between pri-miR-23b~pri-miR-27b) enriched by DGCR8 in HEK293A cells transduced with WT-ADV, DEL-ADV and MUT-ADV for 24 hours and treated with TET (10^{-6} mol/L) for 12 hours.

Supplemental Figure S13. Generation and identification of the G-rich sequence knockout rats. (**A**) Schematic of target site of single guide RNA (sgRNA) targeting the G-rich sequence upstream of miR-24-1. (**B**) Sequencing results of mutant allele in 4 founder rats, revealing different indel mutations in the CRISPR/cas9 target site. Founder #3-6 with an 11 bp deletion was used to generate homozygous rats with loss of the G-rich sequence. (**C**) The whole-genome sequencing of the G-rich sequence knockout homozygous rats.

Supplemental Figure S14. Deletion of G-rich sequence leads to higher expression of miR-24, miR-27b and miR-23b in the NRCMs and NRCFs. (**A-C**) miR-24 (**A**), miR-27b (**B**) and miR-23b (**C**) expression in NRCMs of G4-KO or WT rats. (**D-F**) miR-24 (**D**), miR-27b (**E**) and miR-23b (**F**) expression in NRCFs (Neonatal Rat Cardiac Fibroblasts) of G4-KO or WT rats. Representative data of 7 mice/group are shown (**P*<0.05; ***P*<0.01).

Supplemental Figure S15. Bioinformatics analysis of the targets of miR-24, miR-27b and miR-23b and functional annotation. (**A**) MiRanda, miRDB and psRobot were combined used to search for the targets of three miRNAs, followed by the Kyoto Encyclopedia of Genes and Genomes (KEGG) pathway analysis. (**B**) The KEGG pathway analysis of these targets of three miRNAs. (**C**) MiR-24 binding sites on the 3'UTRs of human and rat JP2 mRNA sequences. (**D**) MiR-27b

binding sites on the 3'UTRs of human and rat EHD3 mRNA sequences. (**E**) MiR-23b binding sites on the 3'UTRs of human and rat SMAD5 mRNA sequences.

Supplemental Figure S16. Deletion of the G4 results in the reduction of mRNA targets of miR-24, miR-27b and miR-23b in rat heart. (**A**) JP2 mRNA expression of cardiac tissues in G4-KO rats compared with WT rats. (**B**) Representative western blots for JP2 protein expression of cardiac tissue in G4-KO and WT rats (left). Quantification of the relative protein level of JP2 in G4-KO rats compared with WT rats (right). (**C**) EHD3 mRNA expression of cardiac tissue in G4-KO rats compared with WT rats. (**D**) Representative western blots for EHD3 protein expression of cardiac tissue in G4-KO and WT rats (left). Quantification of the relative protein level of EHD3 in G4-KO rats compared with WT rats (right). (**E**) SMAD5 mRNA expression of cardiac tissues in G4-KO rats compared with WT rats. (**F**) Representative western blots for SMAD5 protein expression of cardiac tissues in G4-KO and WT rats (left). Quantification of the relative protein level of SMAD5 in G4-KO rats compared with WT rats (right). Representative data of 7 mice/group are shown (*P<0.05; **P<0.01; ***P<0.001).

Supplementary Figure S1

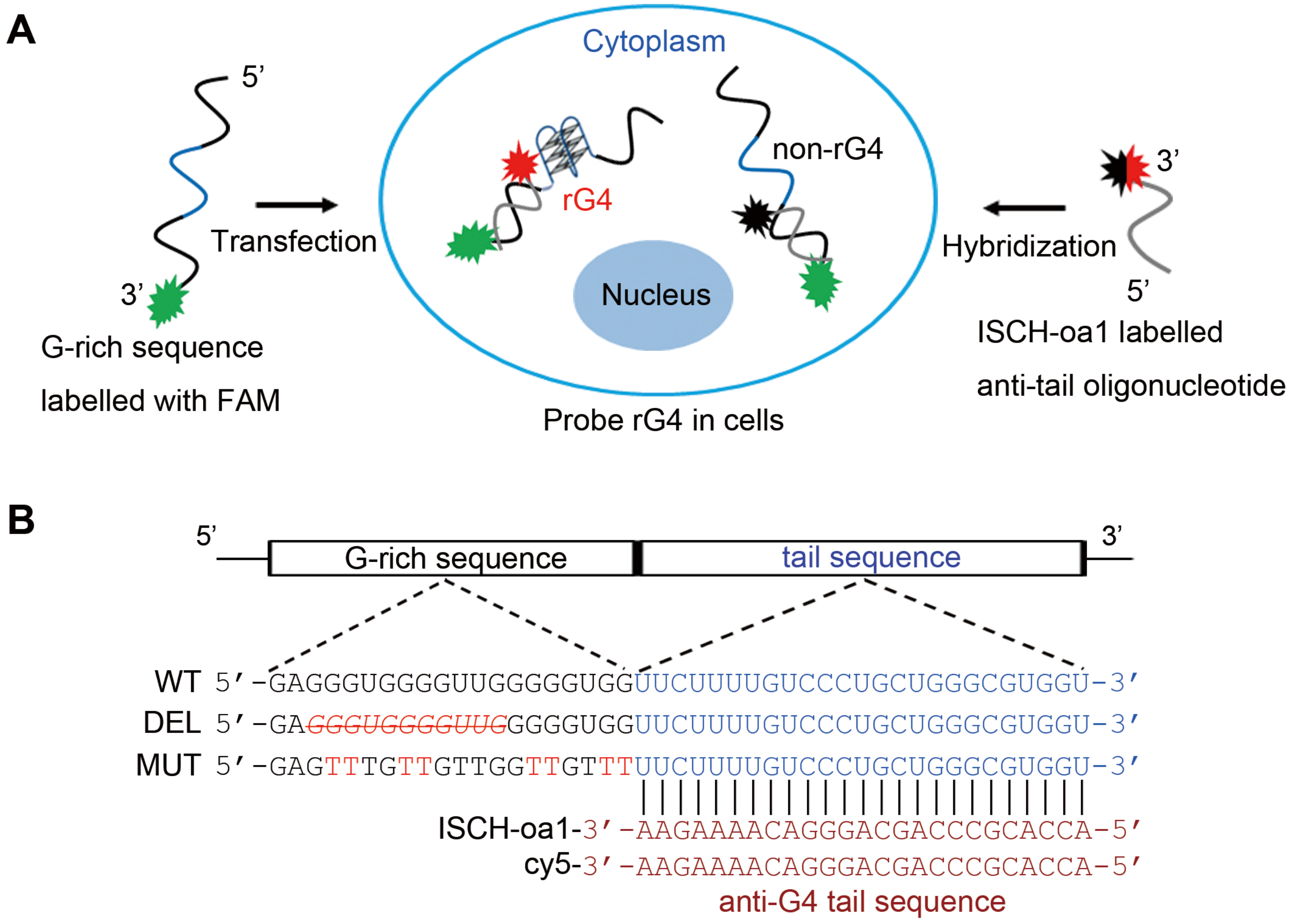

Supplementary Figure S2

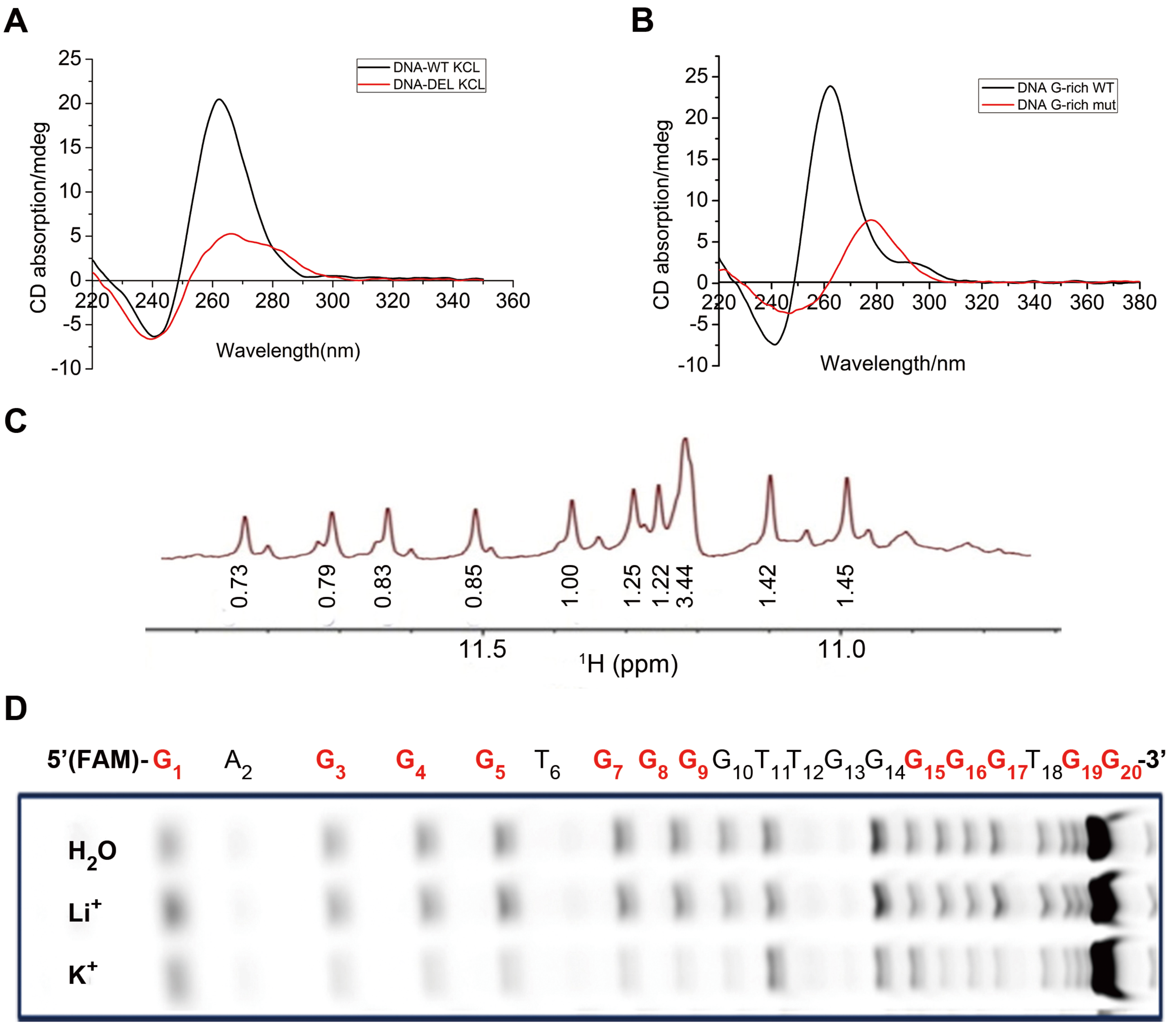

Supplementary Figure S3

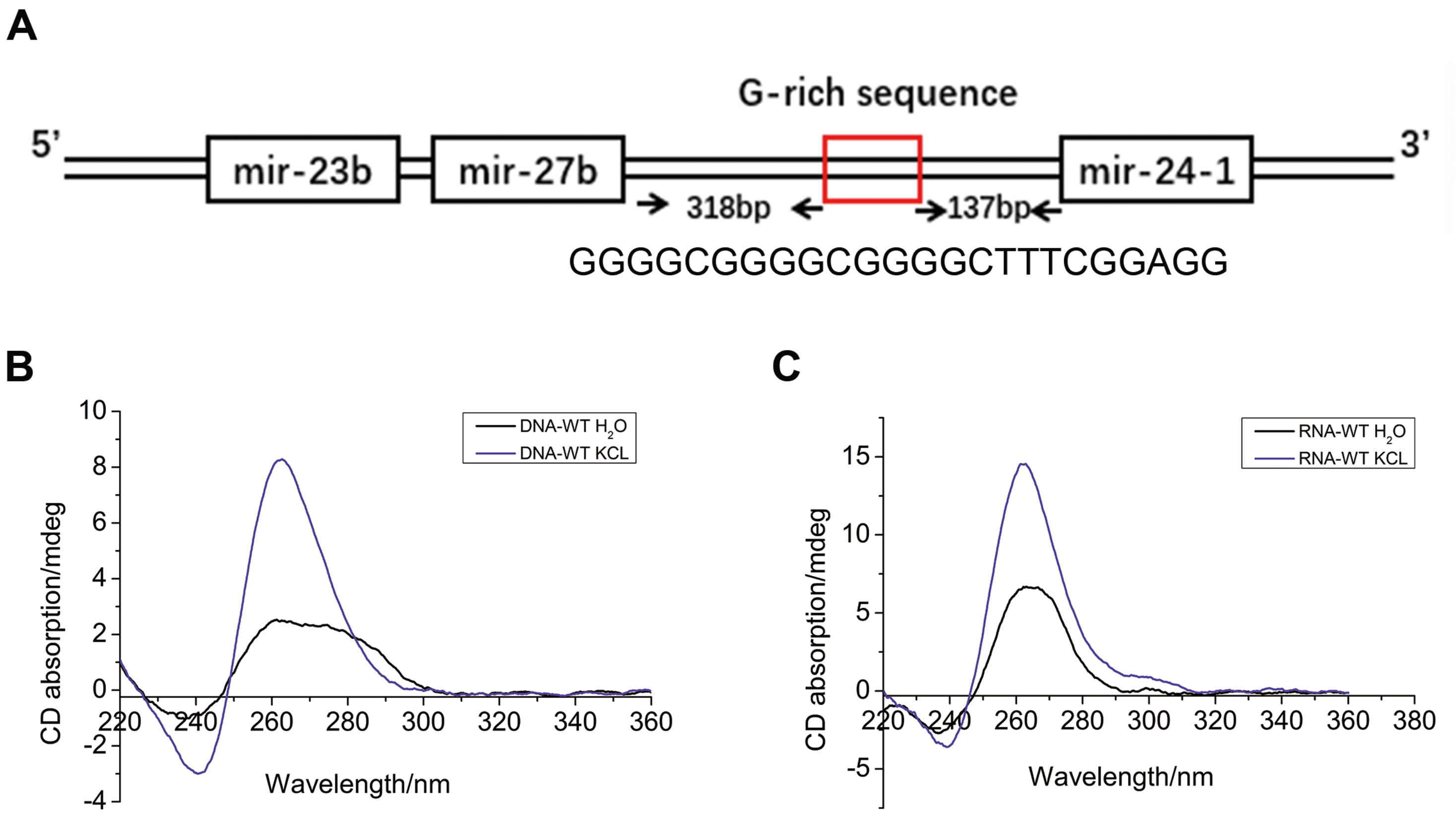

Supplementary Figure S4

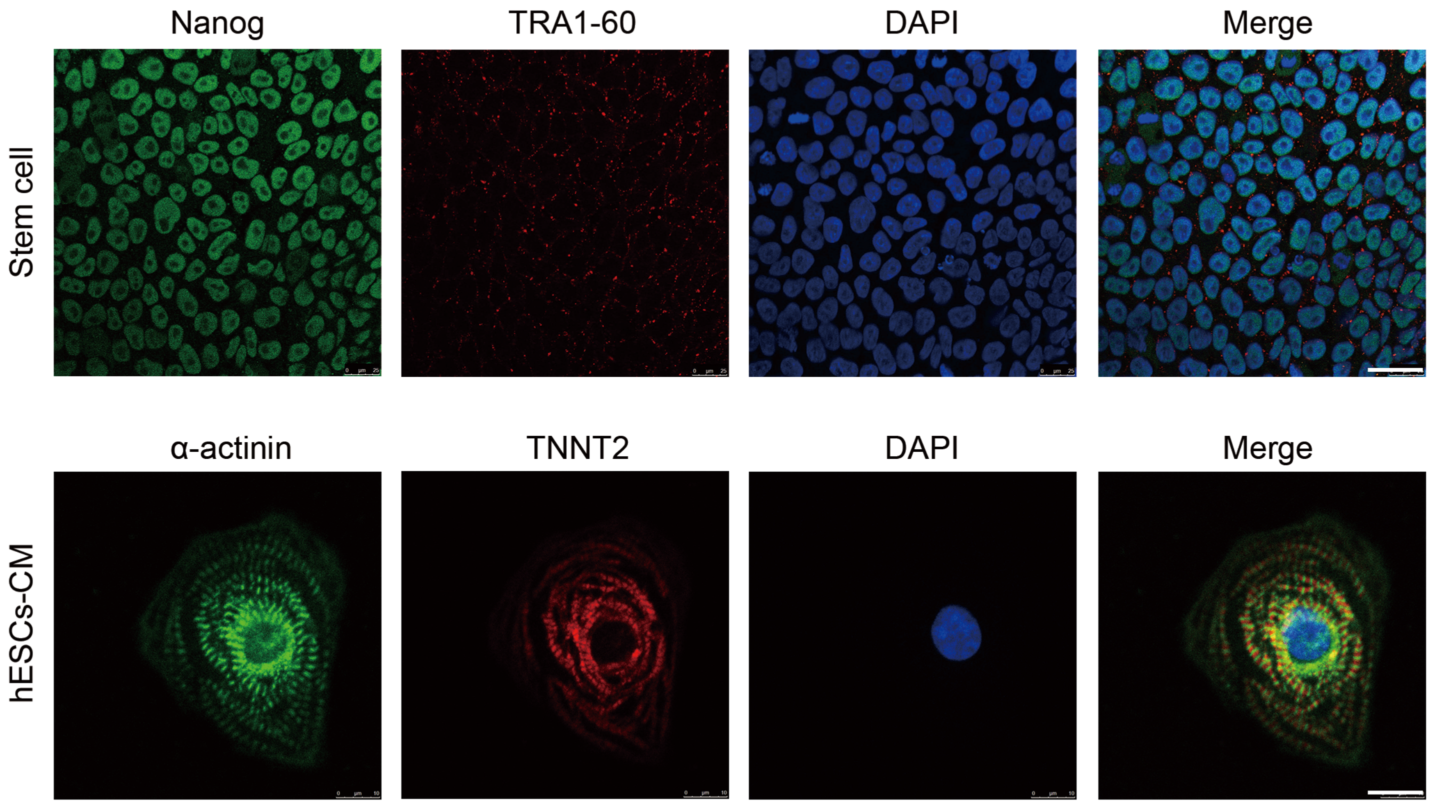

Supplementary Figure S5

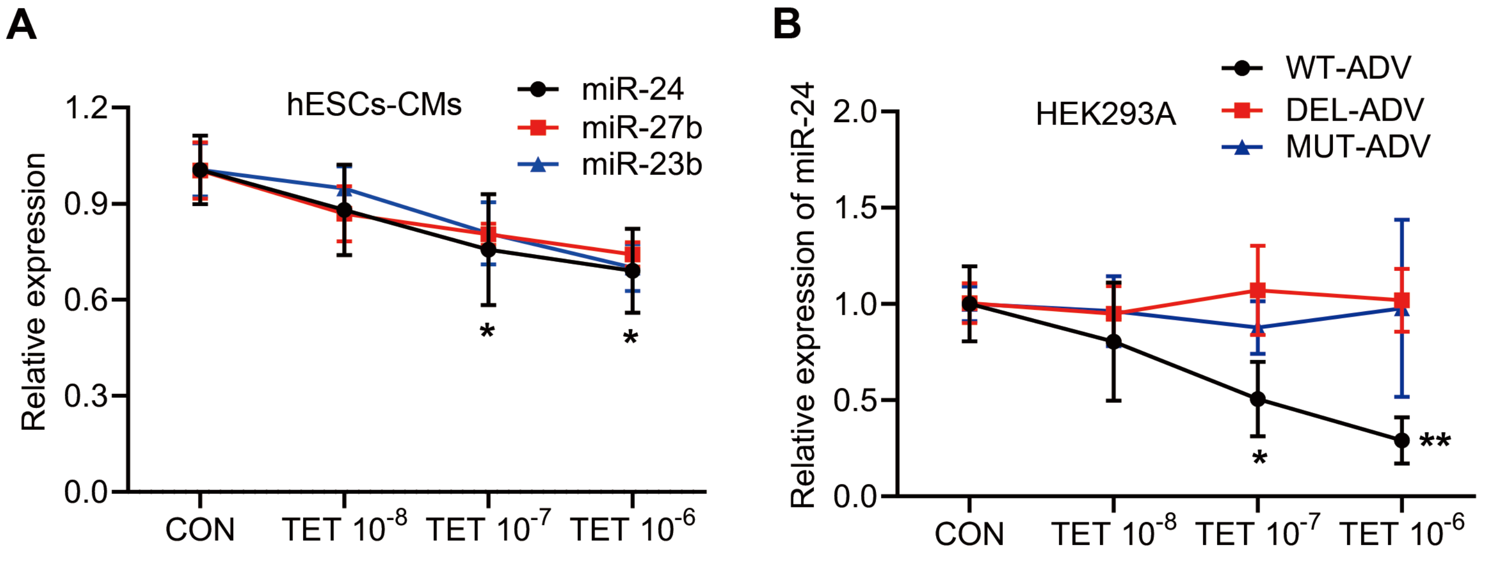

Supplementary Figure S6

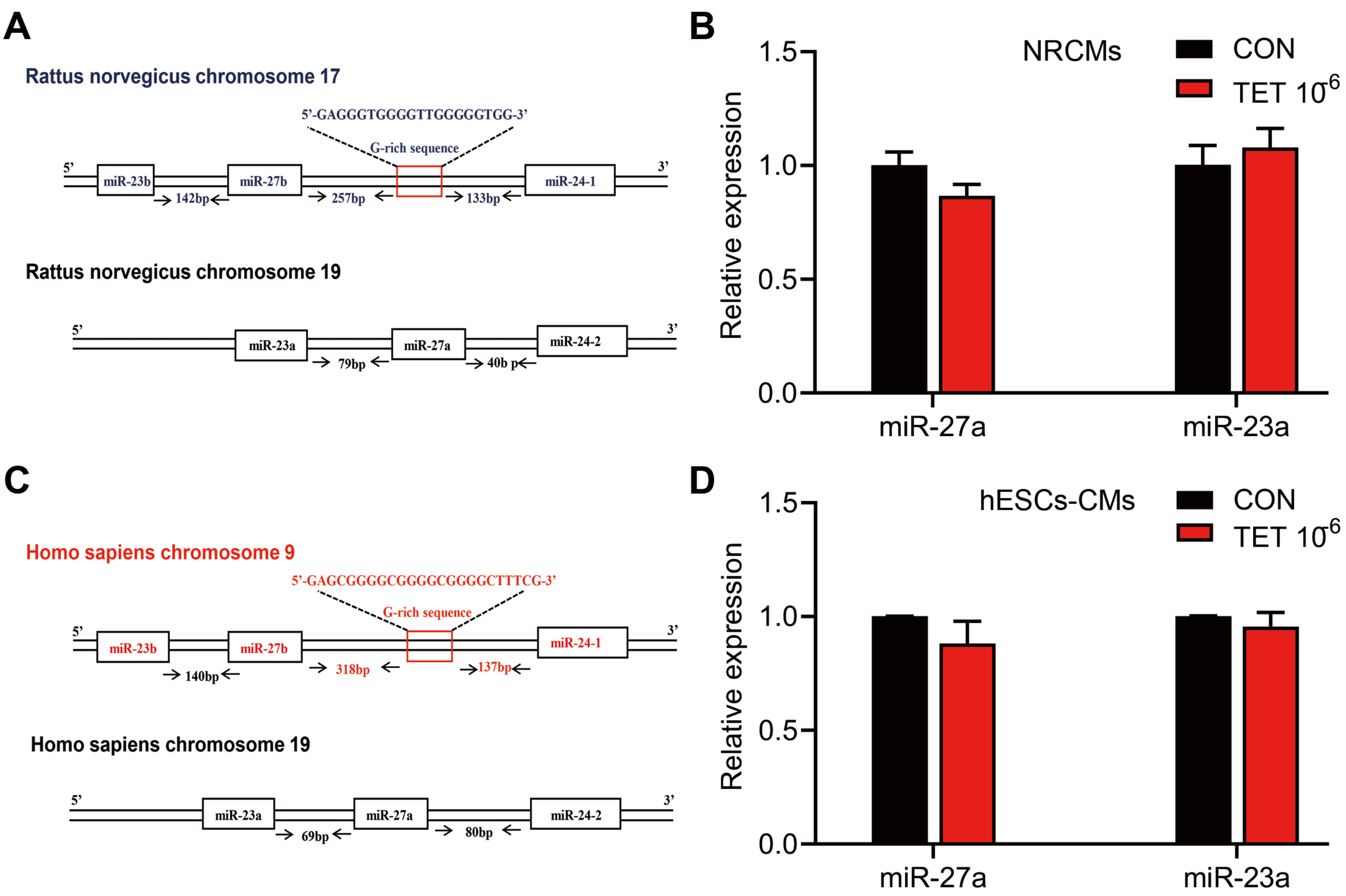

Supplementary Figure S7

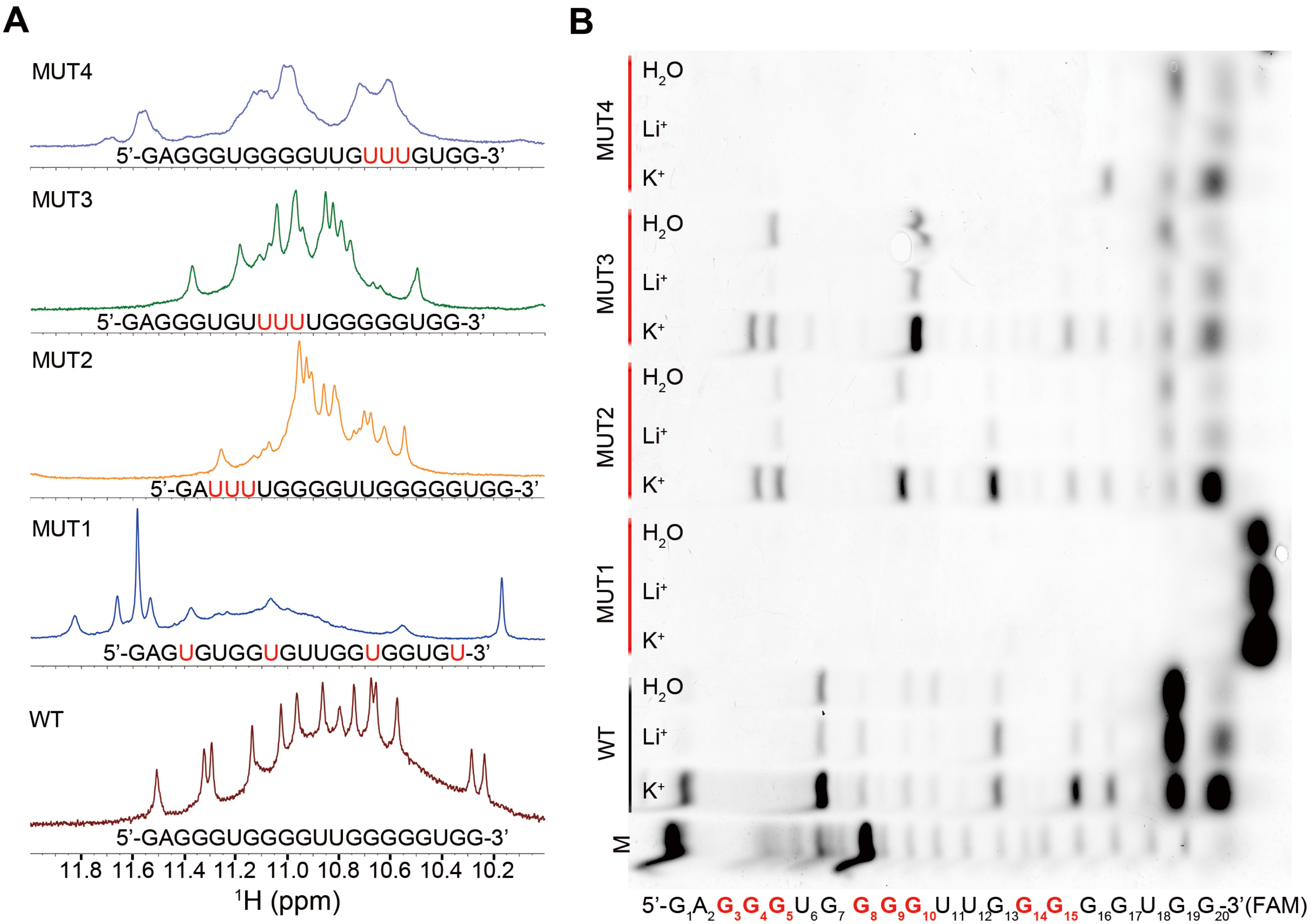

Supplementary Figure S8

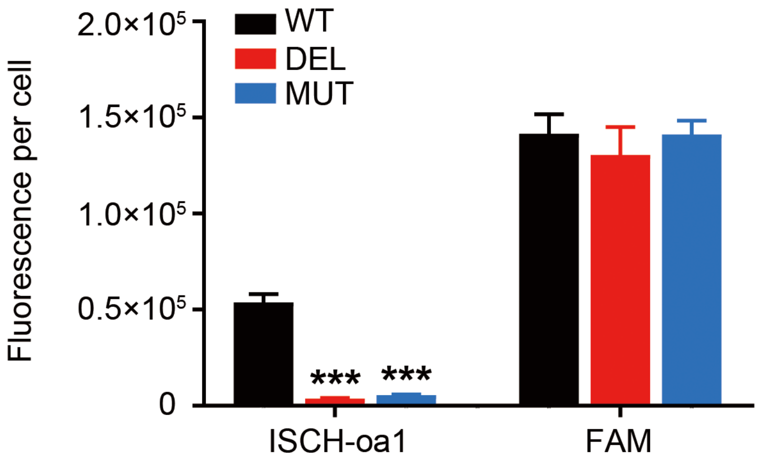

Supplementary Figure S9

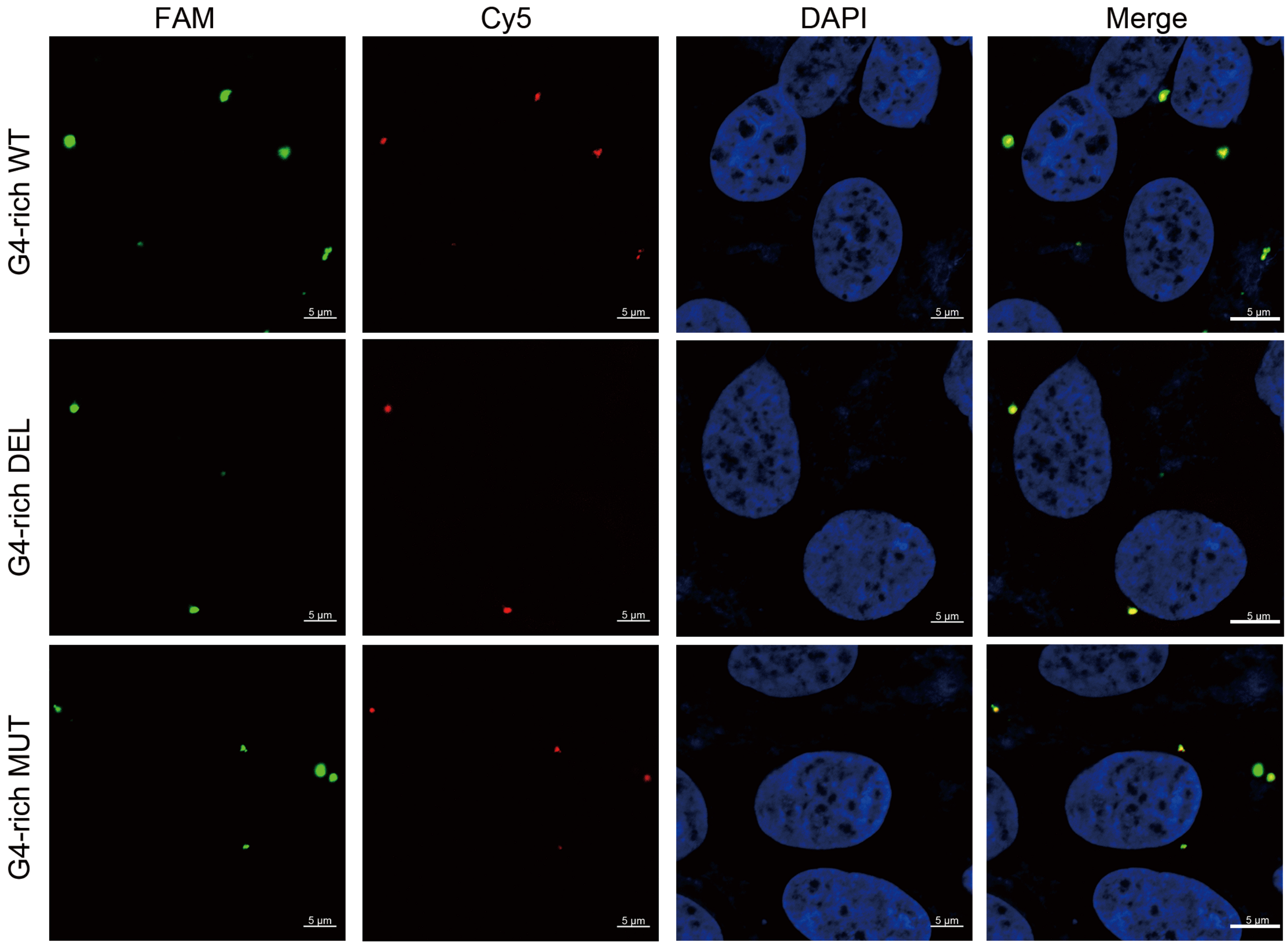

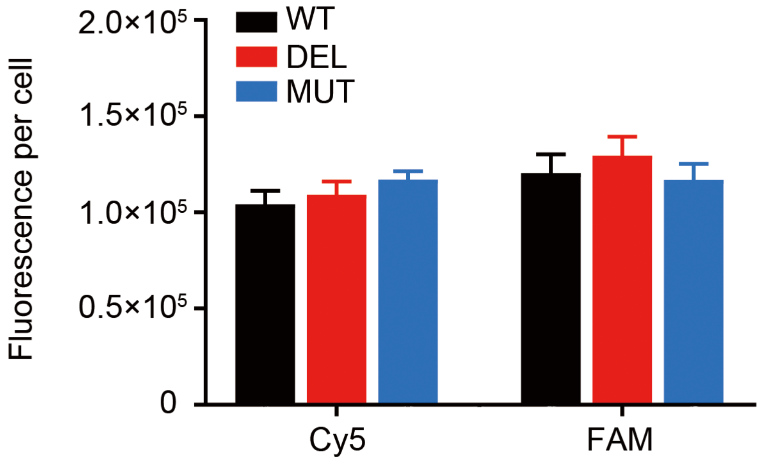

Supplementary Figure S10

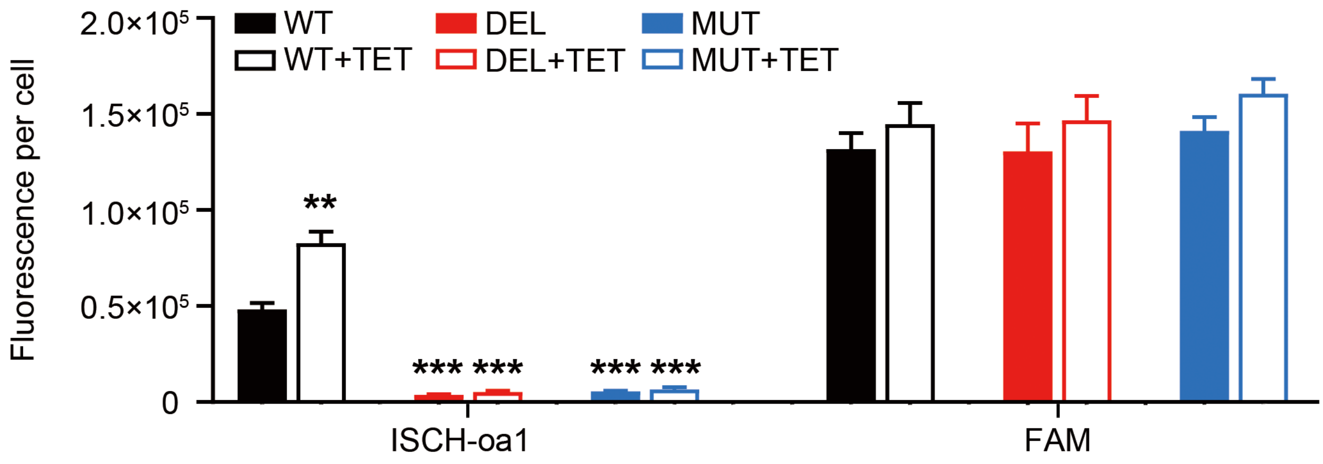

Supplementary Figure S11

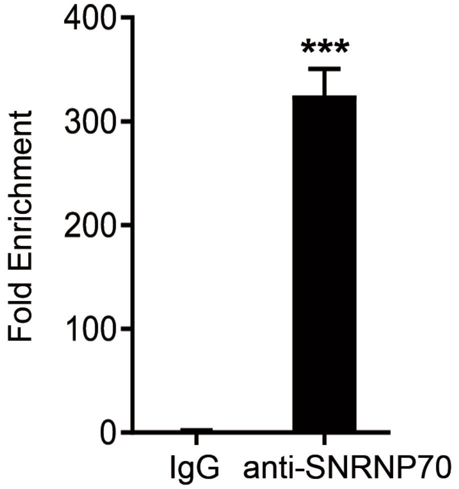

Supplementary Figure S12

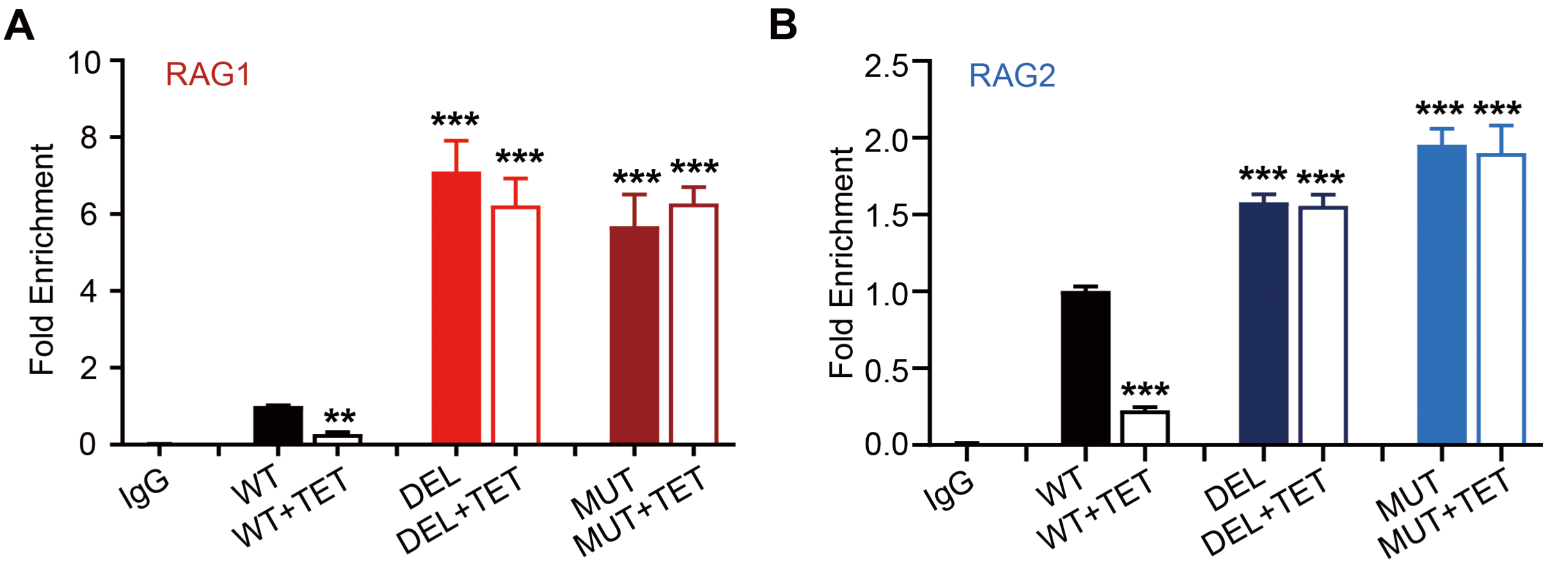

Supplementary Figure S13

A

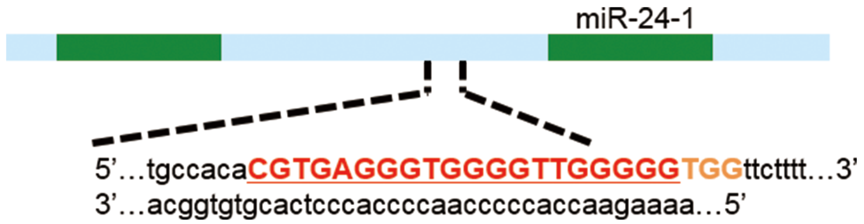

B

**WT**	TGCCACACGTGAGGGTGGGGTTGGGGGTGGTTCTTTT	0
**#3-6**	TGCCACACGTGAGGG:::::::::::::::::::::::::::GTGGTTCTTTT	Δ11
**#3-7 (allel1)**	TGCCACACGTGAGGGTGG::::::TTGGGGGTGGTTCTTTT	Δ2
**#3-7 (allel2)**	TGCCACACGTGAGGGT::::::::::::::TGGGGGTGGTTCTTTT	Δ5
**#3-9**	TGCCACACGTGAGGGTGG::::::::::::::::GGGTGGTTCTTTT	Δ6
**#3-12**	TGCCACACGTGAGGGTGG::::::TTGGGGGTGGTTCTTTT	Δ2

C

chrNew9	112028414	D2	CT	*	hete	74	16	14
chrNew9	112060833	D1	A	*	hete	11	28	25
chrNew9	112070255	D11	GGGTGGGGTTG	*	homo	2059	47	47
chrNew9	112071140	D10	CGGGCTCCCG	*	hete	497	50	50
chrNew9	112071196	I1	T	*	hete	625	52	52

Supplementary Figure S14

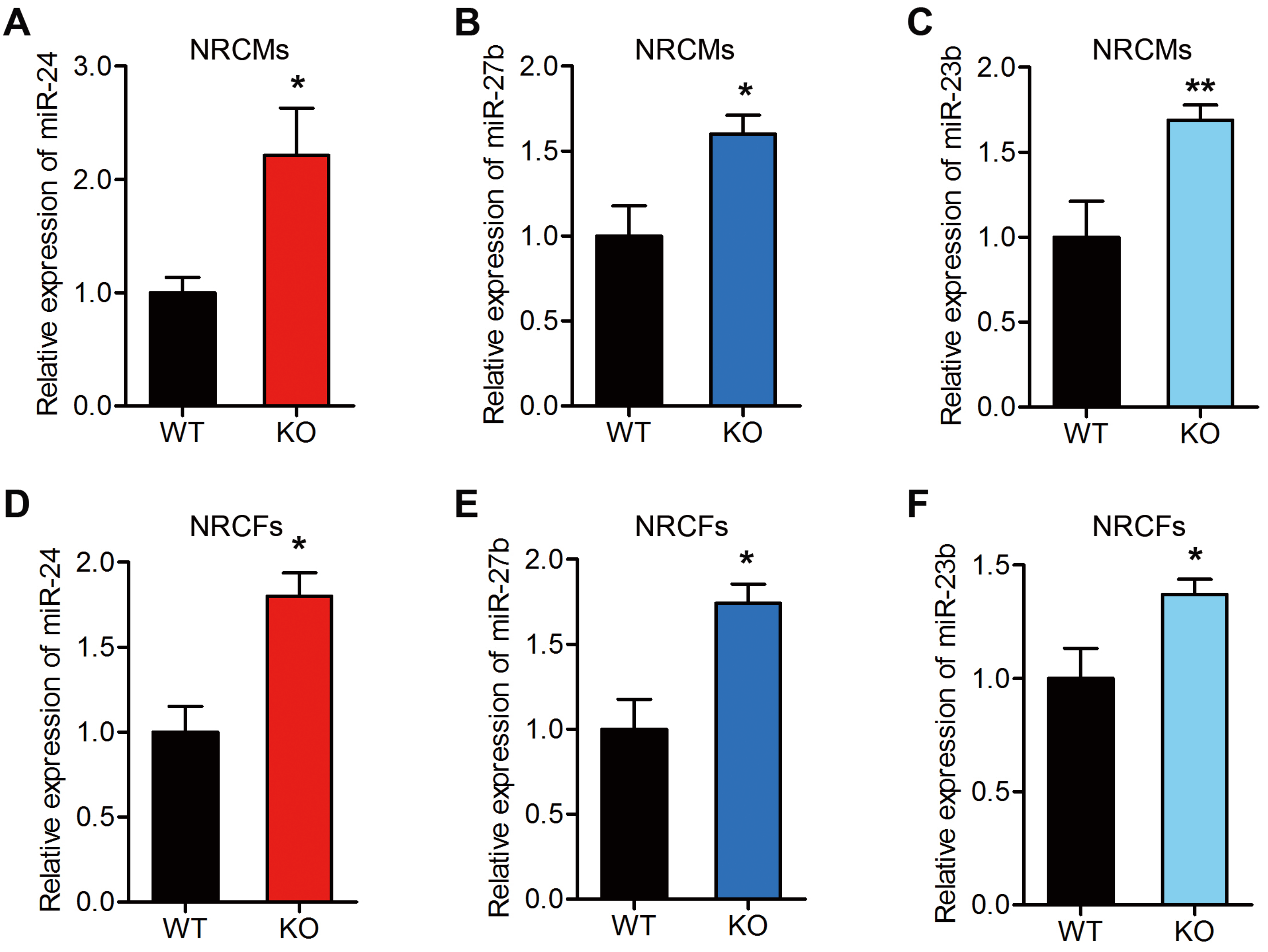

Supplementary Figure S15

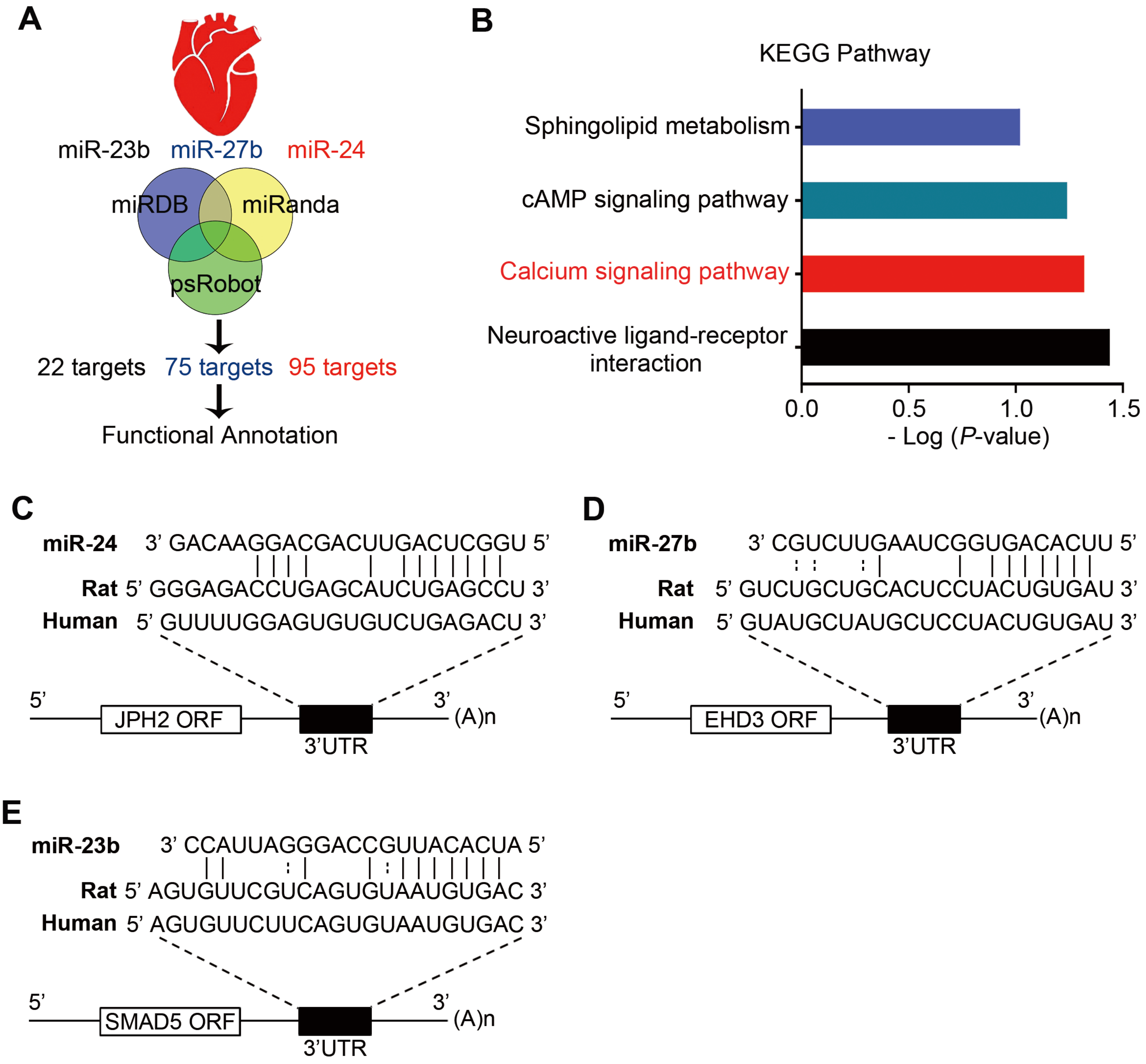

Supplementary Figure S16

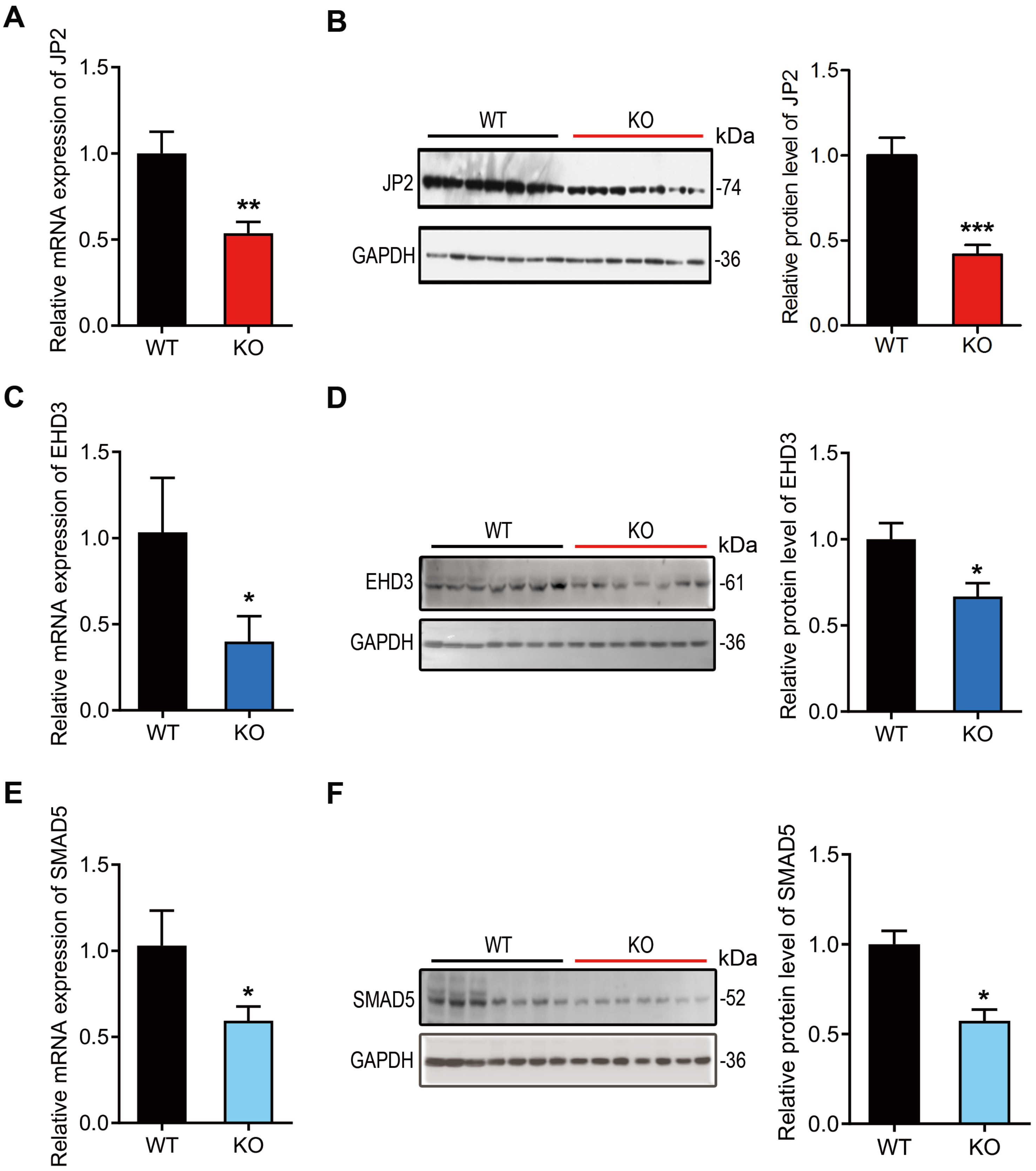

SUPPLEMENTARY TABLES

Supplementary table S1. G-rich Sequences and modified sequences used in this study

Sequence name	Sequence
Rno-DNA WT G-rich seq	5'-GAGGGTGGGGTTGGGGGTGG-3'
Rno-DNA DEL G-rich seq	5'-CACACGTGAGGGGTGGTTCT-3'
Rno-DNA Mut G-rich seq	5'-GAGTTTGTTGTTGGTTGTTT-3'
Has-DNA WT G-rich seq	5'-GGGGCGGGGCGGGGCTTTCGGAGG-3'
Has-RNA Mut G-rich seq	5'-GGGGCGGGGCGGGGCUUUCGGAGG-3'
Rno-RNA WT G-rich seq	5'-GAGGGUGGGGUUGGGGGUGG-3'
Rno-RNA MUT G-rich seq	5'-GAGUUUGUUGUUGGUUGUUU-3'
Rno-RNA MUT1 G-rich seq	5'-GAGUGUGGUGUUGGUGGUGU-3'
Rno-RNA MUT2 G-rich seq	5'-GAUUUUGGGGUUGGGGGUGG-3'
Rno-RNA MUT3 G-rich seq	5'-GAGGGUGUUUUUGGGGGUGG-3'
Rno-RNA MUT4 G-rich seq	5'-GAGGGUGGGGUUGUUUGUGG-3'
FAM-RNA WT G-rich seq	5'-GAGGGUGGGGUUGGGGGUGG-3'-FAM
FAM-RNA MUT1 G-rich seq	5'-GAGUGUGGUGUUGGUGGUGU-3'-FAM
FAM-RNA MUT2 G-rich seq	5'-GAUUUUGGGGUUGGGGGUGG-3'-FAM
FAM-RNA MUT3 G-rich seq	5'-GAGGGUGUUUUUGGGGGUGG-3'-FAM
FAM-RNA MUT4 G-rich seq	5'-GAGGGUGGGGUUGUUUGUGG-3'-FAM
Azido-anti-G4 tail seq	5'-ACCACGCCCAGCAGGGACAAAAGAA-3'-Azido
Cy5-anti-G4 tail seq	5'-ACCACGCCCAGCAGGGACAAAAGAA-3'-Cy5
Transfected RNA WT G-rich seq	5'-GCUGAUGCCACACGUGAGGGUGGGGUUGGGGG UGGUUCUUUUGUCCCUGCUGGGCGUGGUGAACU-3'-FAM
Transfected RNA DEL G-rich seq	5'-GCUGAUGCCACACGUGAGGGGUGGUUCUU UUGUCCCUGCUGGGCGUGGUGAACU-3'-FAM
Transfected RNA MUT G-rich seq	5'-GCUGAUGCCACACGUGAGUUUGUUGUUGGUUGUU UUUCUUUUGUCCCUGCUGGGCGUGGUGAACU-3'-FAM

Supplementary table S2. Primers used for quantitative or semi-quantitative RT-PCR

Primer name	Sequence (5'-3')
miR-24 Forward Primer	TGGCTCAGTTCAGCAGGAACAG
miR-23b Forward Primer	ATCACATTGCCAGGGATTACC
miR-27b Forward Primer	TTCACAGTGGCTAAGTTCTGC
miR-23a Forward Primer	ATCACATTGCCAGGGATTTCC
miR-27a Forward Primer	TTCACAGTGGCTAAGTTCCGC
U6 Forward Primer	CTCGCTTCGGCAGCACA
Rno-JP2-Forward	AGGCGGGTGCCAAGAAGAAG
Rno-JP2-Reverse	CGATGTTCAGCAGGATCACCA
Rno-EHD3-Forward	GCATGAGGATTGGGCCTGAGC
Rno-EHD3-Reverse	GCTGATCCTCTGTTTCTCAC
Rno-SMAD5-Forward	CGTTGGTGGGGAGGTGTATG
Rno-SMAD5-Reverse	CAGACGGTGGTGGGGTGGAA
Rno-PAK4-Forward	GACATCAAGAGTGACTCCATCC
Rno-PAK4-Reverse	ATCACCATTACCCCCAGTGAC
Rno-Rb-Forward	TGGCAGAAATGACTTCTACTC
Rno-Rb-Reverse	CACATCCATGAGACACGA
Rno-E2F1-Forward	CCAGGGAAAGGTGTGAAATCTC
Rno-E2F1-Reverse	GCTCCAAGAAGCGTTTGGT
Rno-GAPDH-Forward	TCCCTCAAGATTGTCAGCAA
Rno-GAPDH-Reverse	AGATCCACAACGGATACATT
Hsa-GAPDH-Forward	CGACCACTTTGTCAAGCTCA
Hsa-GAPDH-Reverse	AGGGGTCTACATGGCAACTG
RAG1-Forward	GCGTGCTGACCACCCTTAAA
RAG1-Reverse	TGCCAGCGACTGTGAAGACA
RAG2-Forward	CCGTGGCTACTTTCTCTCTG
RAG2-Reverse	TTAAAGGGCTGGTGAGCATCTT

Note: The downstream primer of microRNAs is the Oligo dT sequence from miRcute miRNA qPCR reagent kit. The primer of Pre-microRNAs were from QIAGEN and the primer of Pri-microRNAs were from Applied Biosystem.
